# Supplementary material for: PRRT2 Regulates Synaptic Fusion by Directly Modulating SNARE Complex Assembly
Source: Cell Rep. 2018 Jan 29;22(3):820–31. doi: 10.1016/j.celrep.2017.12.056 (PMC5792450; doi:10.1016/j.celrep.2017.12.056)
Supplement: Document S1. Figures S1–S5 [file mmc1.pdf]

Cell Reports, Volume *22*

Supplemental Information

PRRT2 Regulates Synaptic Fusion by Directly Modulating SNARE Complex Assembly

Jeff Coleman, Ouardane Jouannot, Sathish K. Ramakrishnan, Maria N. Zanetti, Jing Wang, Vincenzo Salpietro, Henry Houlden, James E. Rothman, and Shyam S. Krishnakumar

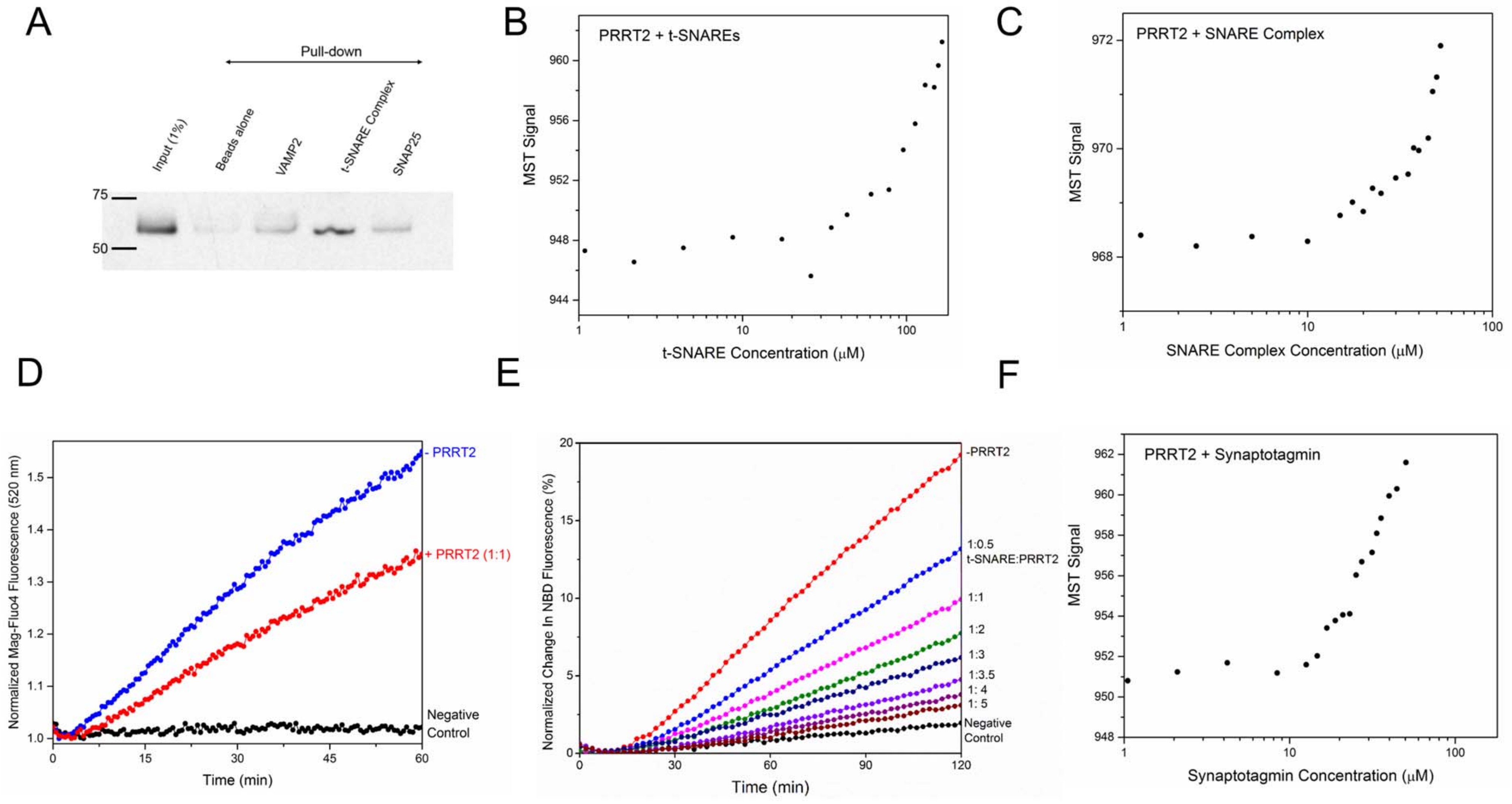

Figure S1. PRRT2 binds individual SNARE proteins to block vesicle fusion

(Related to Figure 1)

PRRT2 has weak interactions with the individual SNARE proteins & Synaptotagmin1. (A) Recombinant PRRT2 was incubated with His^6-tagged individual SNARE proteins (SNAP25 & VAMP2) or assembled t-SNARE (Syntaxin/SNAP25) complex and pulled down using Protein-G resin coated with a monoclonal His antibody. Western blot analysis with PRRT2 antibody revealed the PRRT2 has very weak interaction (1-5% pull-down of the input) with both individual SNARE proteins and assembled t-SNARE complex. PRRT2 interaction with the t-SNAREs (B), SNARE complex (C) and Syt1 (F) was quantified using Microscale Themrophoresis (MST; NanoTemper, Munich, Germany). Themrophoretic properties of PRRT2 labeled at endogenous cysteines with Oregon Green mixed with increasing concentrations of soluble t-SNAREs, pre-assembled SNARE complex and Syt1 was measured using standard treatment capillaries following manufactures instructions. Even at the highest concentration tested (~150 µM for t-SNARE and ~60 µM for SNARE complex and Syt1), the binding was not saturated. But in all cases, we observed binding signal around 25-50 µM underscoring the weak interaction of PRRT2 with the SNAREs and Synaptotagmin. (D) PRRT2 blocks full fusion events. To check this, we adapted a previously described experimental setup (Bello et al., 2016; Shi et al., 2013) using calcium-loaded t-SNARE vesicles fusing with VAMP2-containing nanodiscs, with a calcium-sensitive fluorophore, Mag-Fluo-4 included in the external medium to monitor the release of cargo through a SNARE induced fusion pore. We used larger ~23 nm ApoE-derived nanodiscs containing VAMP2 (vNLPs) and when these vNLP fuses with the calcium-loaded t-SUVs, the Ca^{2+} diffuses through the resulting fusion pore into the exterior buffer, with a consequent increase in Mag-Fluo-4 fluorescence (blue curve). PRRT2 included in the vNLPs at 1:1 molar ratio reduced the Ca^{2+} efflux by ~45% (red curve) confirming that PRRT2 blocks full fusion events. (E) PRRT2 dose analysis using the NBD dequenching liposome fusion assay shows that PRRT2 blocks fusion in a concentration-dependent manner. Increasing amounts of PRRT2 protein were incorporated into t- or v-proteoliposomes, or both in order to reach the indicated ratios of t-SNARE: PRRT2. As a negative control, soluble VAMP2 (CDV) was added in excess to titrate out the t-SNAREs. A representative dose curve is shown and minimum of 3 independent runs were used to estimate the average and deviations shown in Figure 1F.

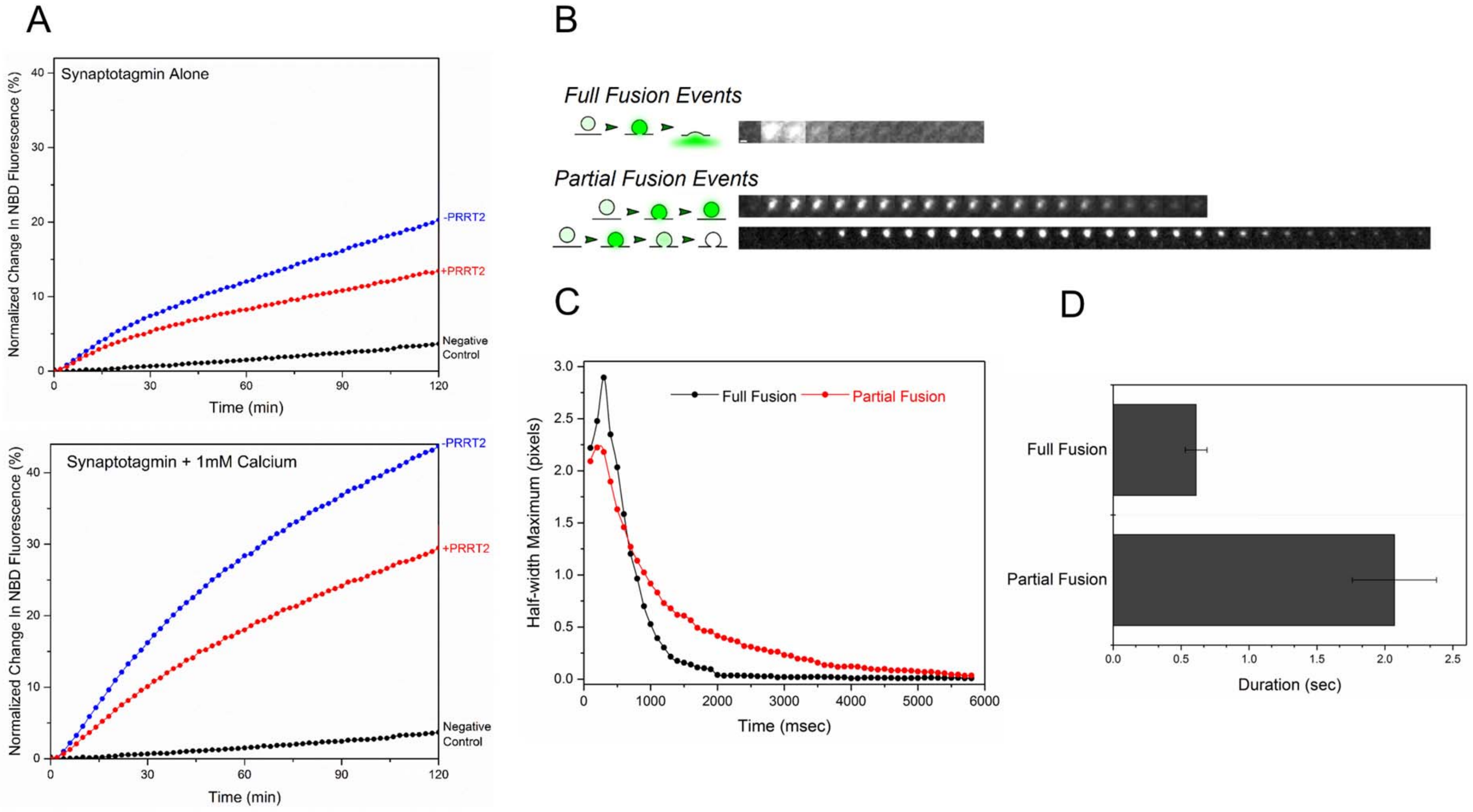

Figure S2. PRRT2 blocks regulated exocytosis under both *in vitro* and *in vivo* conditions.

(Related to Figure 2)

(A) PRRT2 block is realized even in the presence of Synaptotagmin and Ca^{2+}. Lipid mixing assay with PRRT2 and Synaptotagmin reconstituted into t-proteoliposomes (1:1) and v-proteoliposomes (1:4), respectively. The fusion assay was performed with either 0.5 mM EGTA (Synaptotagmin Alone) or 0.5 mM EGTA with 1.5 mM $CaCl_2$ (Synaptotagmin + 1 mM free calcium) in the buffer. In both cases, the inhibitory role of PRRT2 (red curve) was observed and to the same extent indicating that the SNAREs are the primary target of PRRT2 action. Representative fusion curves are shown. (B) Automated analysis of single vesicle exocytosis in PC12 cells using pHluorin under TIRF conditions (A) Time-lapse images of different types of fusion events observed. The fluorescence was recorded at 150 ms per frame under TIRF conditions and cropped to create a time-lapse image. The full fusion events (top) show a diffusive halo and a rapid diffusion of the content, while vesicles undergoing partial fusion (bottom) maintain their shapes and slowly fade due to quenching, slow diffusion through the fusion pore or by leaving the TIRFM area (kiss-and-run events) (C) The fluorescence signal from the vesicle secretion are automatically analyzed by fitting a gaussian with the initial sudden signal increase is set as time zero to align the different events. The average of the half-width versus time shows different dynamics of the different fusion events, with a slower signature for partial fusion events (red), as compared to the faster decay for full-fusion events (black). (D) The comparison of the average duration time (n = 25 cells) for different mode of fusion observed. The duration of an event is defined as the time it takes for the fluorescence to come back to background level signal for a given vesicle. The duration of partial fusion event are substantially (2-3 fold) slower compared to the full-fusion events.

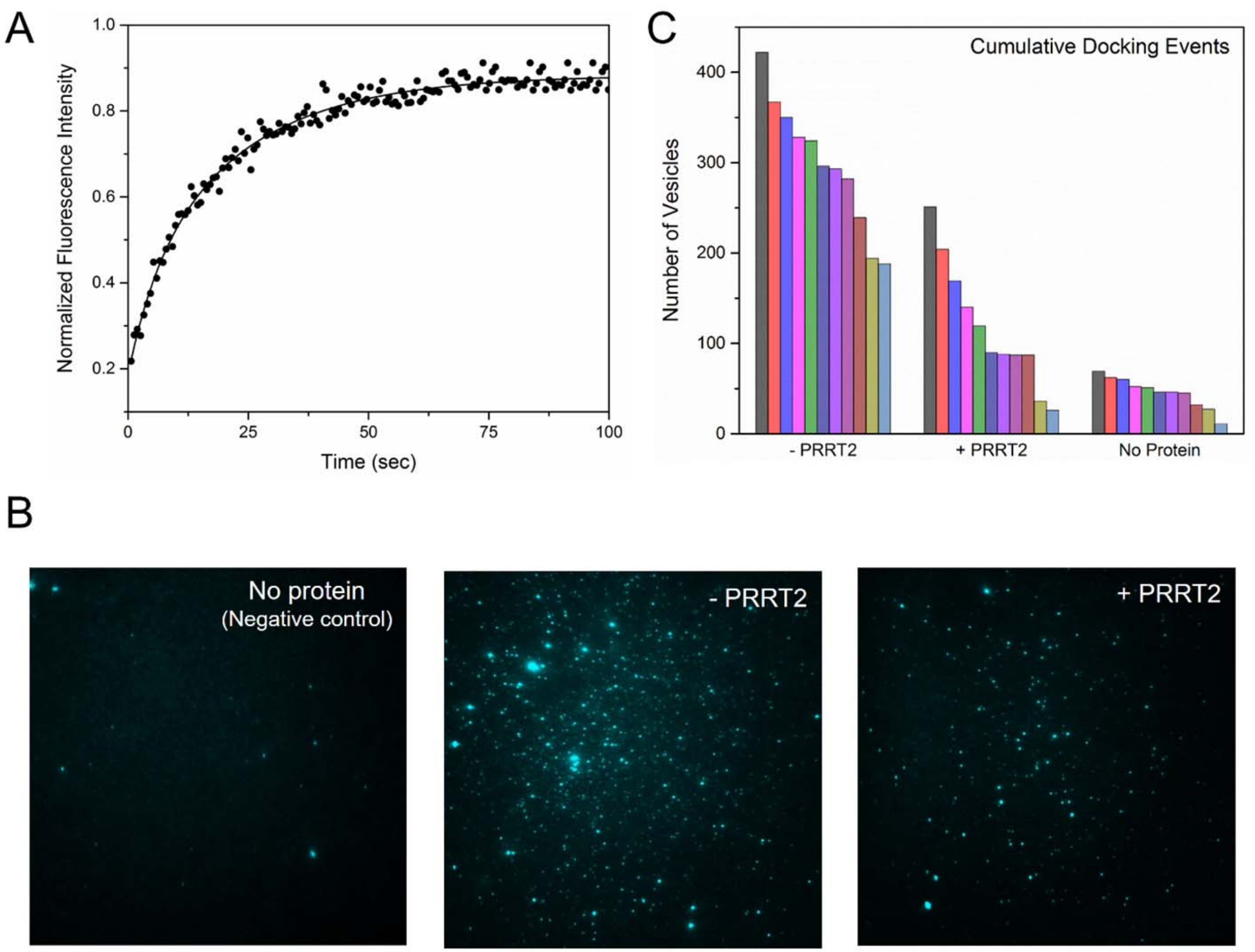

Figure S3. PRRT2 hinders SNARE-mediated docking of individual vesicles to supported bilayer (Related to Figure 3)

The single vesicle docking analysis was carried out under TIRFM conditions, using the ATTO647 dye introduced in the v-liposomes to track the individual vesicles. (A) The fluidity of the t-SNARE containing supported planar bilayer used in the docking analysis was tested by FRAP experiments using the NBD-fluorescence included in the supported bilayer. A representative fluorescence curve (A) shows a rapid recovery following the photo-bleaching consistent with a mobile and fluid bilayer. (B) Representative images of the docked vesicles showing the potent ability of PRRT2 to block SNARE-mediated docking of individual vesicles. (C) Cumulative graph of the number of vesicles docked under various conditions tested. Each color bar denotes an independent experiment carried out with a new supported bilayer. Despite the variability in the total number of vesicles docked between different trials typically introduced by the variability between the supported, bilayers, the trend observed was consistent. For the same concentration of SUVs, there was very little to no docking when VAMP2 was excluded and PRRT2 included in the v-SUVs nearly halved the number of vesicles docking to the supported bilayer.

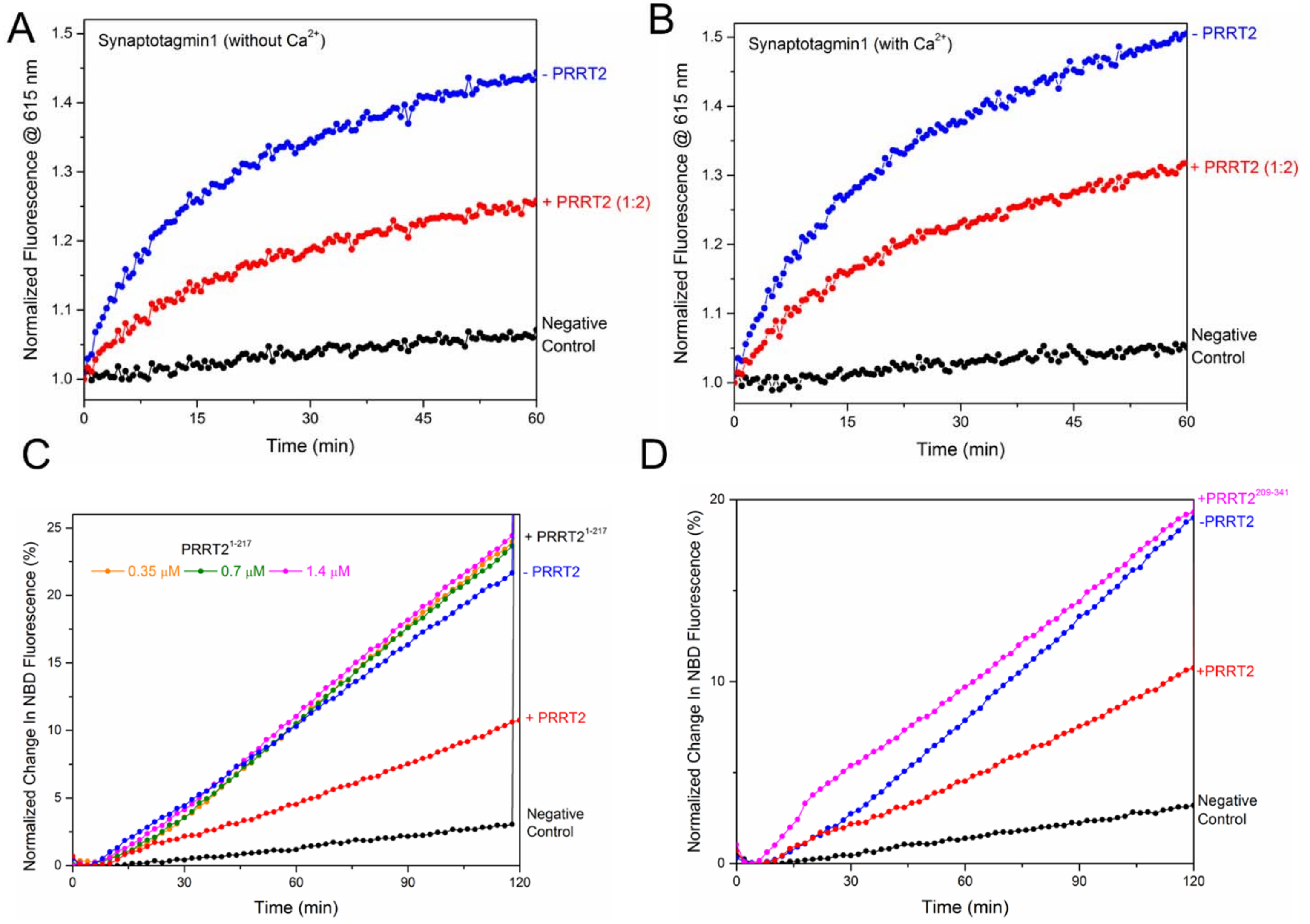

Figure S4. N-terminal Proline-rich domain binds and modulates SNARE Complex Assembly (Related to Figure 4)

(A-B) PRRT2 acts exclusively on the SNARE complex to block fusion. PRRT2 ability to impede the assembly of the N-terminal portion of SNARE complex monitored by FRET between Oregon Green labeled t-SNARE (SNAP25 residue 20) and Texas Red labeled VAMP2 (residue 28) is unaltered by the inclusion of Synaptotagmin1 without Ca^{2+} (A) or with 1 mM free Ca^{2+} (B). Syt1 and Ca^{2+} increases the overall rate and extent of the N-terminal assembly as compared to the SNAREs alone (Figure 4A), but it still inhibited by ~50% when PRRT2 is included in the t-SNARE vesicle at 2:1 PRRT2:t-SNARE ratio. This data confirms that PRRT2-Syt1 interaction is not functionally relevant and SNAREs are the primary target of PRRT2. (C-D) The N- and C-terminal domains of PRRT2 alone are not enough to confer inhibition. (C) The N-terminal portion of PRRT2 (amino acids 1-217) was added to the liposome fusion assay in varying amounts (0.35-1.4 µM) (D) The C-terminal portion (amino acids 209-341) was reconstituted into lipids along with t-SNARE proteins. In both cases, the separate portions of PRRT2 by themselves are unable to inhibit fusion.

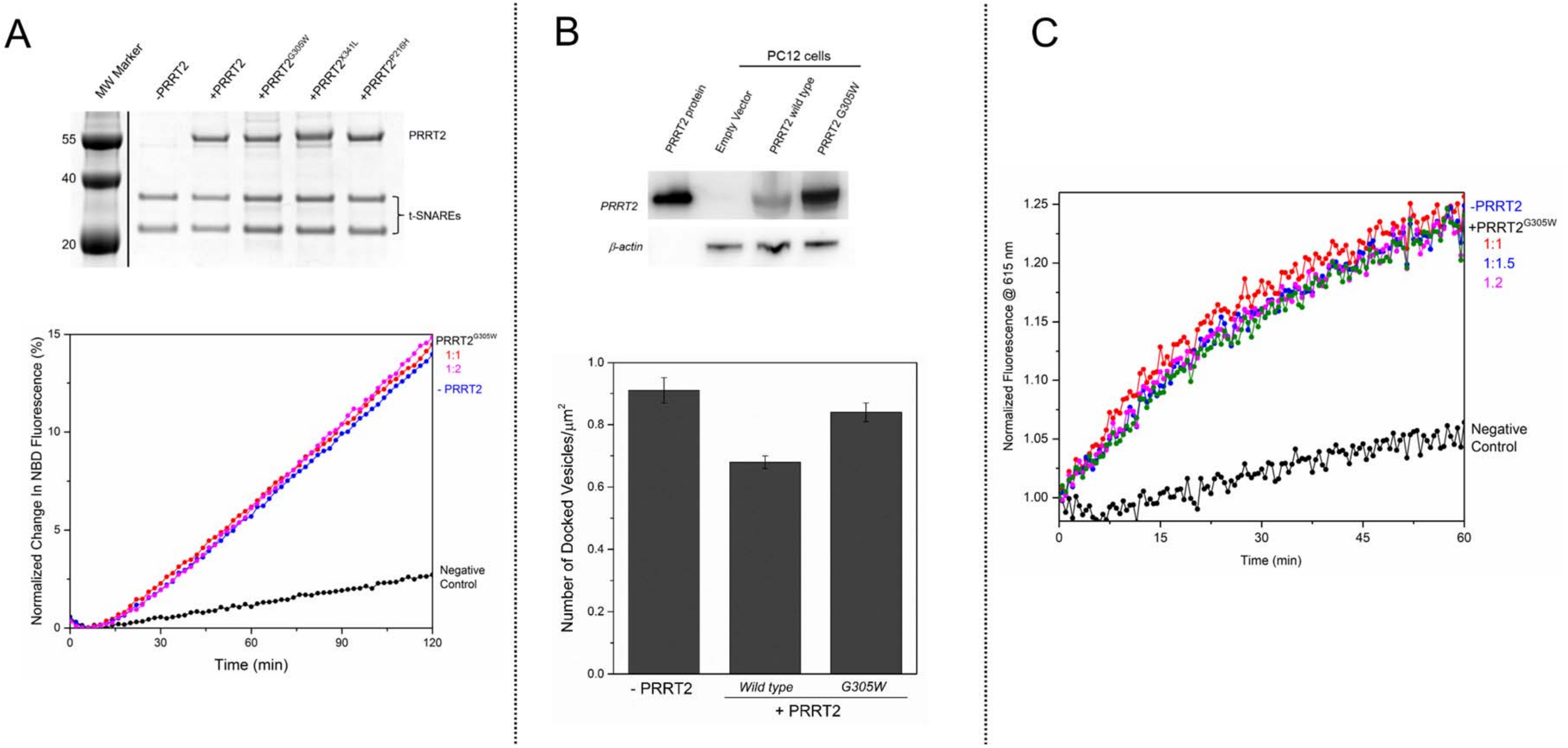

Figure S5. *In vitro* and *In vivo* functional analysis of PKD-associated mutations in PRRT2

(Related to Figure 5)

(A) Coomaisse stained SDS-PAGE analysis of PRRT2 mutants (G305W, X341L & P216H) reconstituted into t-SNARE liposomes. Levels of t-SNAREs were normalized between different samples for liposome fusion assay shown in Figure 5B (top). Dose response PRRT2 mutant $G^{305}W$ in liposome fusion assay at a t-SNARE: PRRT2 ratio of 1:1 and 1:2 shows that the loss of function is not reversed at higher concentrations (bottom). The average and standard deviations from three independent trials are shown in Figure 5C. (B) Characterization of PRRT2 WT and G305W mutant expression and vesicle docking in PC12 cells. Western blot analysis of PC12 cells transfected with PRRT2 WT or G305W showing the lack of endogenous PRRT2 and overexpression of the PRRT2 constructs. A ß-actin was used as a loading control (top). The number of docked vesicles in either wild type or G305W cells as measured by TIRF microscopy following NH_4Cl treatment of the VAMP-pHluorin marker were comparable suggesting that the loss of function phenotype observed in the fusion analysis (Figure 5D) is not due to expression or docking defects (bottom). (C) Monitoring the N-terminal assembly of the SNARE complex using FRET between Oregon Green labeled t-SNARE (SNAP25 residue 20) and Texas Red labeled VAMP2 (residue 28) shows that the G305W mutation disrupts the SNARE modulatory function of PRRT2. Loss of SNARE inhibitory function was observed even at high PRRT2: t-SNARE ratios highlighting the irreversible nature of the G305W mutation. Representative FRET curves are shown and the average and standard deviations on 3-4 independent trials are presented in Figure 5F.

REFERENCES

Bello, O.D., Auclair, S.M., Rothman, J.E., and Krishnakumar, S.S. (2016). Using ApoE Nanolipoprotein Particles To Analyze SNARE-Induced Fusion Pores. Langmuir *32*, 3015-3023.

Shi, L., Howan, K., Shen, Q.T., Wang, Y.J., Rothman, J.E., and Pincet, F. (2013). Preparation and characterization of SNARE-containing nanodiscs and direct study of cargo release through fusion pores. Nat Protoc *8*, 935-948.
