# Supplementary material for: PRRT2 Regulates Synaptic Fusion by Directly Modulating SNARE Complex Assembly
Source: Cell Rep. 2018 Jan 29;22(3):820–31. doi: 10.1016/j.celrep.2017.12.056 (PMC5792450; doi:10.1016/j.celrep.2017.12.056)
Supplement: Document S2. Article plus Supplemental Information [file mmc2.pdf]

Cell Reports

Article

PRRT2 Regulates Synaptic Fusion by Directly Modulating SNARE Complex Assembly

Graphical Abstract

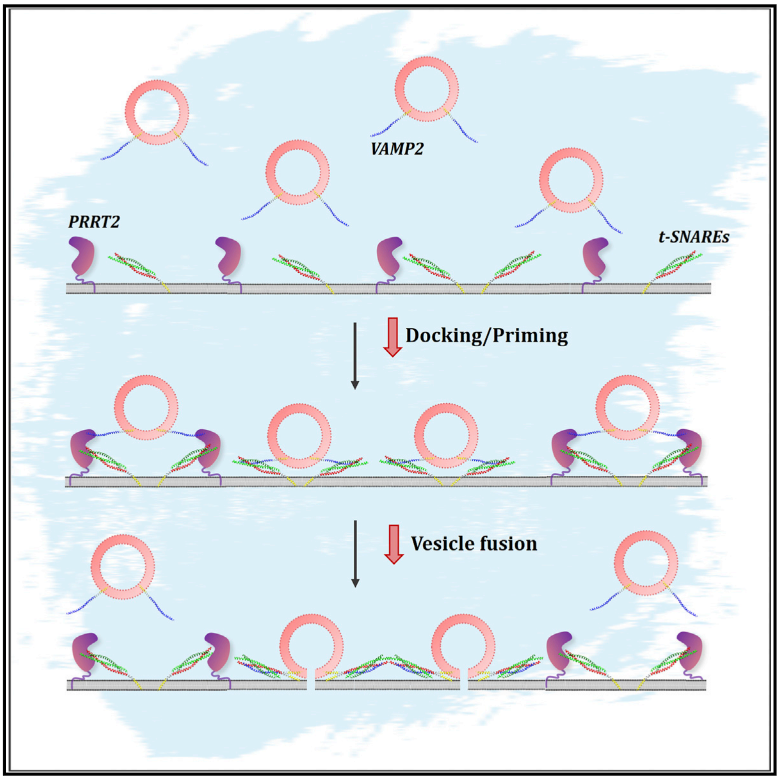

Highlights

- N-terminal proline-rich domain of PRRT2 selectively blocks SNARE complex assembly
- PRRT2 moderates the synaptic vesicle docking/priming process
- Paroxysmal dyskinesia-associated mutations in *PRRT2* disrupt SNARE-modulatory function

Authors

Jeff Coleman, Ouardane Jouannot, Sathish K. Ramakrishnan, ..., Henry Houlden, James E. Rothman, Shyam S. Krishnakumar

Correspondence

james.rothman@yale.edu (J.E.R.),
shyam.krishnakumar@yale.edu (S.S.K.)

In Brief

PRRT2 is linked to several paroxysmal neurological disorders. Coleman et al. identify a crucial role for PRRT2 as a regulator of the synaptic vesicle priming process as it directly binds and influences SNARE complex assembly. A disease-related mutation in PRRT2 disrupts this function, revealing a possible molecular mechanism underlying pathogenesis.

Coleman et al., 2018, Cell Reports *22*, 820–831
January 16, 2018 © 2017 The Author(s).
https://doi.org/10.1016/j.celrep.2017.12.056

CellPress

OPEN ACCESS
CellPress

Cell Reports
Article

PRRT2 Regulates Synaptic Fusion by Directly Modulating SNARE Complex Assembly

Jeff Coleman,[1] Ouardane Jouannot,[1,4] Sathish K. Ramakrishnan,[1,4] Maria N. Zanetti,[2] Jing Wang,[1] Vincenzo Salpietro,[3] Henry Houlden,[3] James E. Rothman,[1,2,*] and Shyam S. Krishnakumar[1,2,5,*]
[1]Department of Cell Biology, Yale University School of Medicine, New Haven, CT 06520, USA
[2]Department of Clinical and Experimental Epilepsy
[3]Department of Molecular Neuroscience, Institute of Neurology
University College London, London WC1N 3BG, UK
[4]These authors contributed equally
[5]Lead Contact
*Correspondence: james.rothman@yale.edu (J.E.R.), shyam.krishnakumar@yale.edu (S.S.K.)
https://doi.org/10.1016/j.celrep.2017.12.056

SUMMARY

Mutations in proline-rich transmembrane protein 2 (PRRT2) are associated with a range of paroxysmal neurological disorders. PRRT2 predominantly localizes to the pre-synaptic terminals and is believed to regulate neurotransmitter release. However, the mechanism of action is unclear. Here, we use reconstituted single vesicle and bulk fusion assays, combined with live cell imaging of single exocytotic events in PC12 cells and biophysical analysis, to delineate the physiological role of PRRT2. We report that PRRT2 selectively blocks the *trans* SNARE complex assembly and thus negatively regulates synaptic vesicle priming. This inhibition is actualized via weak interactions of the N-terminal proline-rich domain with the synaptic SNARE proteins. Furthermore, we demonstrate that paroxysmal dyskinesia-associated mutations in *PRRT2* disrupt this SNARE-modulatory function and with efficiencies corresponding to the severity of the disease phenotype. Our findings provide insights into the molecular mechanisms through which loss-of-function mutations in *PRRT2* result in paroxysmal neurological disorders.

INTRODUCTION

An array of mutations (e.g., non-sense, frameshift, missense) in the gene encoding proline-rich transmembrane protein 2 (PRRT2) are linked to a wide group of paroxysmal disorders, including paroxysmal kinesigenic dyskinesia (PKD), benign familial infantile seizures, infantile convulsions with choreoathetosis, and episodic ataxia (Gardiner et al., 2015). To date, more than 1,500 individuals with 70 different *PRRT2* mutations have been reported (Ebrahimi-Fakhari et al., 2015). However, 78% of patients described so far carry the same frameshift insertion (c.649dupC), which leads to a premature stop codon and nonsense-mediated decay (Chen et al., 2011; Gardiner et al., 2015; Wang et al., 2011). Biallelic (homozygous and compound heterozygous) mutations in *PRRT2* have also been reported in a small number of PKD patients, typically associated with a more severe movement disorder phenotype, learning difficulties, and only partial response to carbamazepine (Delcourt et al., 2015; Gardiner et al., 2015). Furthermore, PRRT2 knockout (KO) and loss-of-function mutant mice display pleiotropic paroxysmal phenotypes and faithfully recapitulate the neurological diseases associated with *PRRT2* mutations (Michetti et al., 2017; Tan et al., 2017). These results indicate that pathogenicity of *PRRT2* mutations is likely due to loss of function and a dominant-negative effect.

PRRT2 is a neuron-specific protein, associating mostly with the pre-synaptic area and to a much lower extent in post-synaptic densities (Valente et al., 2016; Valtorta et al., 2016). Acute silencing of PRRT2 during neuronal development causes a decrease in the density of synaptic connections as well as impairments in synaptic transmission (Valente et al., 2016). This hints at a potential role of PRRT2 in maintaining the pre-synaptic structure and function. Furthermore, screening of PRRT2 interactors by complementary methodologies (Lee et al., 2012; Stelzl et al., 2005; Tan et al., 2017; Valente et al., 2016) reveals that PRRT2 binds to the components of synaptic fusion machinery, particularly the SNARE (soluble N-ethylmaleimide-sensitive factor attachment protein receptor) proteins (Lee et al., 2012; Stelzl et al., 2005) and the Ca^{2+} sensors synaptotagmin 1/2 (Valente et al., 2016). This suggests that PRRT2 is likely involved in regulating synaptic vesicle fusion and the ensuing release of neurotransmitters (Valente et al., 2016; Valtorta et al., 2016). However, the complex nature of the cellular environment, compounded by the variable phenotypes and the pleiotropic effects of genetic manipulations, preclude further insight into the molecular mechanisms. Thus, a reductionist approach, in which variability of some of the parameters is restricted, is required to deduce the physiological role of PRRT2 and the underlying molecular mechanisms.

Fusion of synaptic vesicles is mediated by the neuronal SNARE proteins wherein polarized assembly of the vesicle-associated v-SNARE (VAMP2) with its cognate t-SNARE (syntaxin1a/SNAP25) on the target membranes provides the energy to fuse the bilayers and open the fusion pore (Ji et al., 2010; Söllner et al., 1993; Weber et al., 1998). This constitutive process is tightly controlled by several regulatory elements to achieve

 © 2017 The Author(s).
This is an open access article under the CC BY license (http://creativecommons.org/licenses/by/4.0/).

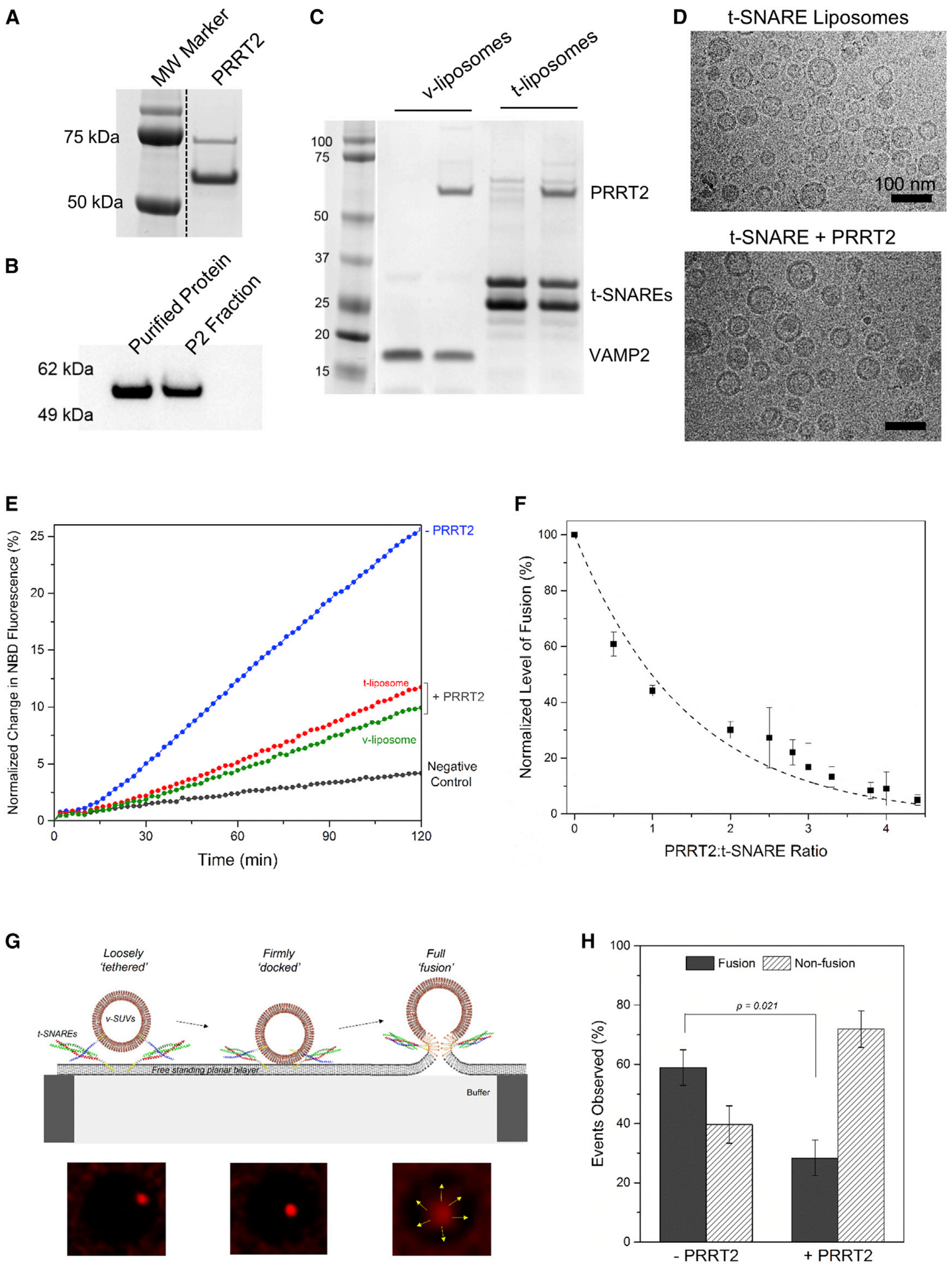

Figure 1. PRRT2 Inhibits Synaptic SNARE-Mediated Fusion under Reconstituted Conditions

(A) Purity of the recombinant PRRT2 purified using bacterial expression setup is confirmed using Coomassie-stained SDS-PAGE analysis.

(B) Identity of the purified protein is verified using western blot analysis with a PRRT2 antibody. The native protein from the P2 synaptosomal fraction was used as positive control.

(C) Purified PRRT2 reconstituted into VAMP2- or t-SNARE-containing liposomes using detergent dilution and dialysis method is analyzed using Coomassie-stained SDS-PAGE analysis.

(legend continued on next page)

Ca^{2+}-synchronized rapid neurotransmitter release (Südhof, 2013; Südhof and Rothman, 2009). Reconstituted *in vitro* fusion assays, typically monitoring lipid mixing between compartments, are an important tool in the study of the membrane fusion process (Ji et al., 2010; Kyoung et al., 2013; Weber et al., 1998). This versatile assay, wherein the relevant proteins and co-factors can be added or altered individually, has greatly contributed to our understanding of synaptic fusion (Ji et al., 2010; Kyoung et al., 2013; Weber et al., 1998) and the mechanistic details of synaptic fusion regulators, including synaptotagmin1, complexin, Munc18, and Munc13 (Diao et al., 2012; Lai et al., 2017; Ma et al., 2013; Malsam et al., 2012; Shen et al., 2007).

We use this reconstituted setup, at both bulk and single-vesicle levels, to establish the functional capabilities of PRRT2 and to obtain a detailed understanding of the structure-function relationship. We report that the N-terminal proline-rich region on PRRT2 selectively blocks synaptic SNARE-mediated fusion by preventing the initial engagement of the SNARE proteins and thus modulates the vesicle priming process. Fluorescence imaging analysis of neuroexocytosis in PC12 cells confirms that this function extends to regulated exocytosis. Remarkably, some PKD-associated PRRT2 mutations disrupt the SNARE-modulatory function, revealing a possible mechanism that might underlie the associated paroxysmal phenotype.

RESULTS

PRRT2 Inhibits Synaptic SNARE-Mediated Fusion of Liposomes

Informed by the recent findings that PRRT2 may interact with the core synaptic fusion machinery (Lee et al., 2012; Tan et al., 2017; Valente et al., 2016), we used a defined fusion system (Weber et al., 1998) to identify the molecular role of PRRT2 at the synapse. To this end, we purified the full-length PRRT2 as a GST-tagged protein using a bacterial expression system. The purity of the isolated PRRT2 protein was checked using Coomassie-stained SDS-PAGE analysis (Figure 1A), and the identity was verified using an anti-PRRT2 immunoblot (Figure 1B). Both showed a single band at ~60 kDa, comparable with the native PRRT2 protein in the brain synaptosomal (P2) fraction (Figures 1A and 1B). Subsequently, we examined if there is a direct molecular interaction between purified PRRT2 and the SNARE proteins. His^6 pull-down assays revealed a weak but comparable binding of purified PRRT2 with both the VAMP2 and the t-SNARE complexes (Figure S1A). We used microscale thermophoresis to quantify this interaction. Titration of the t-SNARE or the assembled SNARE complex in solution exhibited a dose-response curve, but binding was not saturated even at the highest concentrations tested (~150 μM for t-SNARE and ~60 μM for the SNARE complex) (Figures S1B and S1C). So, PRRT2 interacts directly with individual SNARE proteins and the SNARE complex but has a low affinity ($K_d \geq 50$ μM) under soluble conditions

To assess the functional relevancy of this interaction, we used the reconstituted, lipid-mixing assay based on NBD (N-[7-nitro-2-1,3-benzoxadiazol-4-yl])-RHO (lissamine rhodamine B) energy transfer (Ji et al., 2010; Weber et al., 1998) and as such incorporated the purified PRRT2 into SNARE-containing liposomes at a defined PRRT2/SNARE ratio (Figure 1C). Cryoelectron microscopy, applied as a quality control of the proteoliposome reconstitution, showed that the majority of vesicles in both the control and PRRT2-containing samples were unilamellar, with diameters of 70–100 nm and no structural abnormalities (Figure 1D).

The synaptic SNAREs were reconstituted at physiologically relevant surface densities, and because PRRT2 localizes primarily to the pre-synaptic plasma membrane, it was first introduced into t-SNARE liposomes at the same density (~50 copies of each protein per vesicle). As shown in Figure 1E, PRRT2 lowered both the rate and the extent of SNARE-mediated fusion and reduced the total fusion after 2 hr by ~50%. This was a dynamic effect and did not require pre-binding to the SNAREs, as we observed similar inhibition of fusion (~54%) with PRRT2, at the same density as before, incorporated into v-SNARE liposomes (Figure 1E). We also tested and confirmed that PRRT2 blocks full fusion with a content release assay (Bello et al., 2016; Shi et al., 2013) using calcium-loaded t-vesicles fusing with v-nanodiscs and a calcium-sensitive fluorophore, Mag-Fluo-4, included in the external medium (Figure S1D). Furthermore, we analyzed fusion by systematically varying the PRRT2/t-SNARE ratio. Consistent with defined molecular interaction, PRRT2 inhibited fusion in a concentration-dependent manner and exhibited a classical dose-response curve, with half-maximal response corresponding to about one copy of PRRT2 for each t-SNARE and near complete block of fusion at two or three copies of PRRT2 per t-SNARE

(D) Cryoelectron microscopy analysis of reconstituted t-liposomes shows that PRRT2 incorporation does not alter the size or other physical attributes of the liposomes. Representative micrographs of t-SNARE liposomes with or without PRRT2 (1:1) incorporated are shown.

(E) PRRT2 introduced in either the v- or t-liposomes inhibited synaptic SNARE-mediated fusion of liposomes monitored by NBD dequenching assay. Negative control with soluble cytoplasmic domain of VAMP2 (CDV) added in excess to titrate out the t-SNAREs shows that PRRT2 is not inherently fusogenic. Representative fusion curves are shown.

(F) PRRT2 displays a typical dose-response curve corresponding to a unique bio-molecular interaction. The dose-response curve was constructed from the maximal fusion levels observed for varying PRRT2 concentrations. Dotted line shows a single exponential decay fit, which estimates the half-maximal response at a 1:1 PRRT2/t-SNARE ratio. Average values and SDs from a minimum of three independent experiments are shown.

(G) Schematic of the single-vesicle fusion analysis. Fluorescent-labeled lipid (ATTO647-PE) included in v-SNARE-containing liposomes (v-SUV) enables us to track the association and fusion of single vesicles with the free-standing planar bilayer using confocal microscopy. Typically, the vesicle appears in the field of view when it loosely tethers to the bilayer (left), progresses to firmly dock concomitant with an increase in the fluorescence signal (middle), and then proceeds to fuse (right), evidenced by the radial diffusion of the fluorescent lipids in the bilayer after transfer due to membrane fusion. Our automated software enables the tracking of individual vesicles and classify the different stages, leading to fusion.

(H) PRRT2 reduces the fraction of vesicles that proceed to fuse in the single-vesicle analysis. In the absence of PRRT2, ~60% of all observed vesicles proceed to full fusion. PRRT2 introduced in the v-SUVs significantly reduces (~50%) the fraction of fused vesicles. Average values and SDs from a minimum of three independent experiments are shown.

(Figures 1F and S1E). In all cases, control experiments confirmed that PRRT2 is not inherently fusogenic and the cognate SNAREs are required to catalyze fusion under all conditions.

To dissect this further, we analyzed the effect of PRRT2 on rapid fusion of single vesicles containing VAMP2 (v-liposome) with planar, free-standing bilayers containing the synaptic t-SNAREs (S.K.R., A. Gohlke, F. Li, J.C., X. Wu, J.E.R., and F. Pincet, unpublished data). At low vesicle concentration, the fluorescent marker (Atto 647 1,2-dioleoyl-sn-glycero-3-phosphoethanolamine [ATTO647-DOPE]) included into the v-liposome allows us to readily observe and quantitate individual vesicles attaching and then fusing with the planar bilayer using standard confocal microscopy (Figure 1G). With the SNAREs alone, ~60% of the vesicles that attach proceeded to fuse, while the remainder (~40%) stayed attached and gradually photobleached or visibly dissociated from the bilayer (Figure 1H). Inclusion of PRRT2 in the v-liposomes significantly reduced the proportion of fusion events, wherein only a minor fraction (~30%) of attached vesicles progressed to fuse (Figure 1H). Noteworthy, the magnitude of fusion inhibition (~50%) in this analysis is comparable with that observed in the bulk fusion assays (Figures 1E and 1F). Taken together, our data indicate that PRRT2 directly binds synaptic SNARE protein to negatively regulate the SNARE-mediated vesicle fusion process.

PRRT2 Blocks Synaptotagmin1/Ca^{2+}-Regulated Fusion of Liposomes

At the synapse, SNARE-catalyzed vesicle fusion is chaperoned by several regulatory elements that confer speed and synchronicity (Südhof, 2013; Südhof and Rothman, 2009). A key regulator is the Ca^{2+} sensor synaptotagmin1 (Syt1), which couples vesicle fusion to Ca^{2+} influx following action potential (Brose et al., 1992; Geppert et al., 1994). In fact, PRRT2 has been predicted to bind Syt1 on the basis of co-immunoprecipitation studies (Valente et al., 2016), and our MST analysis confirmed this, revealing a direct but weak interaction, comparable to the SNARE proteins (Figure S1F). So, we examined if PRRT2 modulates the Syt1-regulated membrane fusion. In a functional reconstitution with full-length Syt1 incorporated into the v-liposomes, PRRT2 (included at a 1:1 ratio to the t-SNAREs) inhibited both the Ca^{2+}-independent and Ca^{2+}-triggered membrane fusion (Figure 2A) and, remarkably, to same extent when Syt1/Ca^{2+} were absent (Figures 2B and S2A). This indicates that the PRRT2 clamping function is realized even under conditions of regulated exocytosis. It further reveals that PRRT2 exerts an irreversible block on fusion, unlike other synaptic regulators such as complexin (Malsam et al., 2012), and does so by selectively acting on the SNARE proteins, independent of other co-factors.

PRRT2 Blocks Ca^{2+}-Regulated Neuroexocytosis in PC12 Cells

To physiologically validate these findings, we examined the effect of PRRT2 on Ca^{2+}-evoked exocytosis in rat adrenal pheochromocytoma (PC12) cells. PC12 cells are widely used as a model for neurosecretion because regulated exocytosis in the PC12 cells is mediated by the neuronal fusion machinery (Burgoyne and Morgan, 1998; Westerink and Ewing, 2008). In addition, the PC12 cell line lacks endogenous PRRT2 and thus provides an ideal background to establish the functional capabilities of PRRT2 in a cellular context (Figure 2C). We used live-cell fluorescence imaging to monitor single exocytotic events by using pHluorin-tagged (luminal) VAMP2 (Figure 2D). We chose VAMP2-pHluorin because it serves as a universal marker for all vesicle types (small clear vesicles, small and large dense core vesicles) in PC12 cells. In our total internal reflection fluorescence (TIRF) microscopy setup (Miesenböck et al., 1998; Ryan, 2001; Toomre, 2012), the pHluorin signal increase upon fusion is automatically detected by a differential analysis of the images and fitted to a Gaussian to classify into full-fusion or partial-fusion events on the basis of its duration and decay characteristics (Sebastian et al., 2006; Toomre, 2012; Xu et al., 2011) (Figure S2B). Expression of PRRT2 in PC12 cells strongly reduced the number of fusion events triggered by potassium chloride (KCl) depolarization in the presence of Ca^{2+} (Figure 2D). Detailed analysis further revealed that PRRT2 inhibits both the full- and partial-fusion events, without altering the ratio between the two modes of fusion (Figure 2D). Thus, PRRT2 plays a vital role in moderating synaptic SNARE-mediated vesicle fusion in the cellular context.

PRRT2 Modulates the Vesicle Priming Process

Vesicular exocytosis involves a series of morphologically and molecularly defined sequential steps wherein vesicles first translocate to and are loosely "tethered" at the plasma membrane (PM), then undergo preparatory molecular reactions termed "priming/docking," which render them fusion-competent and culminate in membrane fusion, either constitutively or when triggered by an external stimulus (Verhage and Sørensen, 2008). So, we looked into which stage(s) of this sequential process is regulated by PRRT2.

Ammonium chloride (NH_4Cl) treatment, which neutralizes the pH and permits visualization of all tethered/docked but unfused vesicles by TIRF microscopy, showed that PRRT2 does not substantially alter the number of vesicles that are at or near (~250 nm) the PM (Figure 3A). This was corroborated by electron microscopy (EM) analysis, which showed that PPRT2 expression had only a modest effect on the density and distribution of the trafficking dense core vesicles (Figure 3B). The minor difference (~10% reduction) in the number of vesicles locating close to the PM (Figures 3A and 3B) cannot explain the robust inhibition of exocytosis by PRRT2 (Figure 2D). However, the EM/TIRF analysis does not have resolution to distinguish between loosely tethered and primed/docked vesicles in the cellular context, so we used a reconstituted setup to investigate in detail.

We used a TIRF microscopy-based assay to examine the effect of PRRT2 on SNARE-mediated docking/priming of vesicles (Karatekin and Rothman, 2012; Xu et al., 2016). To assay for single-vesicle docking, freely diffusing fluorescently (ATTO647-DOPE) labeled v-SNARE liposomes were allowed to react with a t-SNARE-containing planar supported bilayer for 10 min and imaged following an extensive wash to remove any non-specifically bound vesicles. To get an accurate estimate of the docked vesicles, we used VAMP2 protein with mutations in the C-terminal half (L70D, A74R, A81D, and L84D, termed VAMP2-4X) that eliminates fusion activity without impeding the docking process (Krishnakumar et al., 2011, 2013). A control experiment with

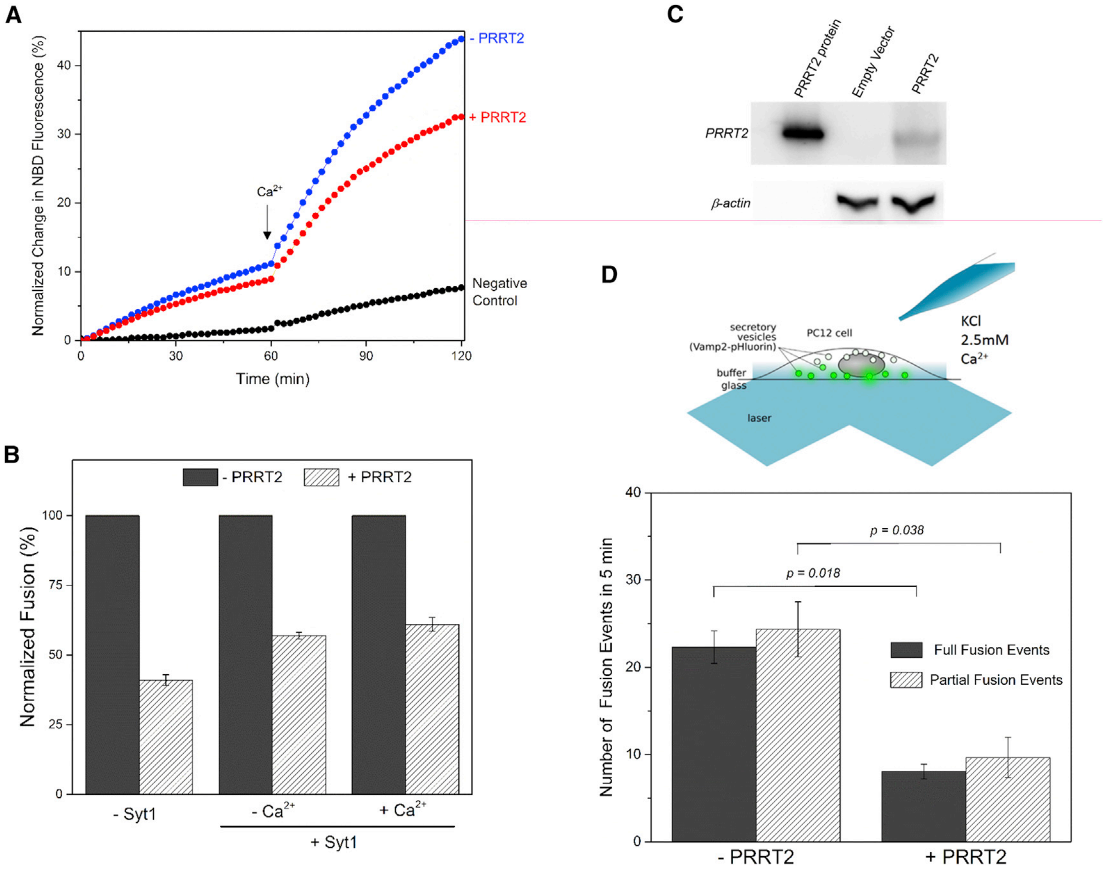

Figure 2. PRRT2 Blocks Ca^{2+}-Regulated Exocytosis under Both Reconstituted and Physiological Conditions

(A) Reconstituted fusion assay with synaptotagmin in the v-SNARE vesicles shows that PRRT2 also blocks synaptotagmin-regulated fusion, and the block is not altered by addition of Ca^{2+}. Representative fusion curves are shown.

(B) Direct comparison of the PRRT2 fusion block under both synaptotagmin-free and synaptotagmin ± Ca^{2+} conditions shows the extent of inhibition (one copy of PRRT2 per t-SNARE) is comparable under all conditions tested, suggesting that SNARE proteins are the primary target for PRRT2. Average values and SDs from three or four independent experiments are shown.

(C) Western blot analysis shows the PC12 cells lack endogenous PRRT2, thus providing a virgin environment to test the functional capabilities of PRRT2 under physiologically relevant conditions.

(D) TIRF microscopy setup used to observe single exocytotic events in PC12 cells transfected with VAMP2-pHluorin (±PRRT2), stimulated by local perfusion of KCl supplemented with 2.5 mM Ca^{2+}. Live cell imaging analysis using TIRF microscopy shows that PRRT2 inhibits evoked fusion. It blocks both partial and full-fusion events, as characterized by their distinctive fluorescence signatures (Figure S2B). Average values and SDs from three or four independent experiments, with a minimum of 50 cells under each conditions, are shown. Statistical significance was established using a Wilcoxon-Mann-Whitney test to account for the non-normal distribution of the data.

protein-free liposomes confirmed that the docking of vesicle under these conditions is strictly dependent on the SNARE protein, and PRRT2 introduced in the v-liposomes greatly reduced (~60%) the number of bound vesicles (Figures 3C and S3). In fact, this impediment in docking could account for the inhibition observed in the single-vesicle analysis (Figure 1H).

Consequently, we assessed PRRT2's role in regulating membrane fusion itself. Using our single-vesicle fusion system (Figure 1G), we found that PRRT2 does not alter the fusion efficiency (Figure 3D) as the fraction of firmly docked vesicle that end up fusing was comparable in the presence and absence of PRRT2 (76% and 84%, respectively). However, PRRT2 slightly slowed down the overall fusion process (Figure 3E). This is apparent in the survival analysis (i.e., fraction of the unfused vesicles as a function of time), wherein the majority of docked vesicles fuse within 1–2 s in the absence of PRRT2 but in its presence stay docked longer, with the majority fusing around 3–4 s (Figure 3E). This slight delay in fusion is likely due to an impediment imposed by PRRT2 on the assembly of the critical number of SNARE complexes required to drive membrane fusion. Thus, our data clearly demonstrate that PRRT2 acts primarily to moderate the priming process, resulting in a reduction of the number of fusion-competent vesicles and has a modest effect on the fusion of pre-docked vesicles.

The Proline-Rich N-Terminal Domain of PRRT2 Impedes SNARE Engagement

SNARE proteins assemble in a polarized fashion, consisting of two sequential steps (Li et al., 2007, 2016). The first step is

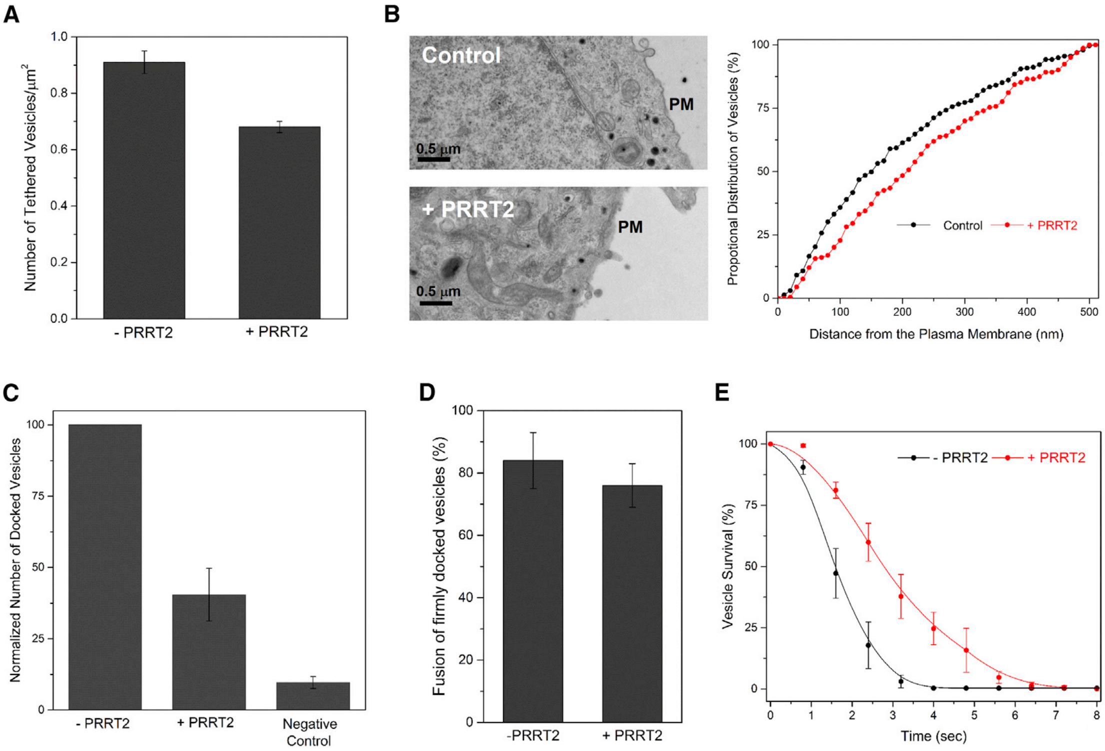

Figure 3. PRRT2 Impedes SNARE-Dependent Docking/Priming of Vesicles

(A) Tethering/docking of the vesicles surveyed by neutralizing the pH in all vesicles with 50 mM ammonium chloride under TIRF conditions shows that PRRT2 expression does not substantially alter the number of vesicles at or near the PM. Average values and SDs from three independent experiments with a minimum of 25 cells per condition are shown.

(B) EM analysis of serial sections of PC12 cells with (left bottom) and without (left top) PRRT2 expression confirms that PRRT2 does not alter the density and distribution of the dense core vesicles. The proportional distribution of the dense core vesicles (dark spots) from the PM is shown. The average distribution from three independent experiments (~25 cells in total) is shown.

(C) TIRF microscopy-based analysis reveals that PRRT2 inhibits the docking of individual v-SNARE liposomes to a t-SNARE-containing planar supported bilayer. To get an accurate estimate of the docked vesicles, VAMP2 protein with mutations in the C-terminal half (L70D, A74R, A81D, and L84D, termed VAMP2-4X) that eliminates fusion activity was used, and the number of firmly docked vesicles was estimated after a 10 min incubation followed by an extensive buffer wash. The number of docked vesicles normalized to the PRRT2-free condition from three to five independent experiments is shown. The error bars indicate the SEM.

(D and E) PRRT2 has a moderate effect on the membrane fusion process. (D) The single-vesicle fusion analysis showing that PRRT2 does not change the efficiency of the fusion process as the percentage of firmly docked vesicles that ultimately fuse is unaltered by the inclusion of PRRT2. However, PRRT2 introduces a slight delay in the fusion of the docked vesicles. The percentage survival curve of docked vesicles (E) reveals that the vesicles containing PRRT2 on an average take longer to fuse compared with control vesicles. Average and SEM from five independent single-vesicle fusion analyses are shown.

the N-terminal assembly, in which the SNAREs zipper approximately two-thirds of the way to completion. This step docks the vesicle to the PM and induces a structural optimization in the SNAREs required for fusion (Li et al., 2007, 2014, 2016). The second step is the assembly of the C-terminal end of the SNAREs, which brings the membranes closer and provides energy for fusion (Li et al., 2007, 2014; Melia et al., 2002). We used a fluorescence resonance energy transfer (FRET) assay, with fluorescent probes introduced along the SNARE proteins, to directly track PRRT2's effect on complex assembly (Krishnakumar et al., 2013). As shown in Figure 4A, PRRT2 lowers the FRET signal between Oregon green and Texas red probes introduced at the N termini of SNAP25 and VAMP2, respectively, in a concentration-dependent manner. We obtained indistinguishable results from FRET probes introduced at the C termini (Figure 4B), implying that PRRT2 essentially impedes the initial engagement of the SNARE proteins. Inclusion of Syt1, with or without Ca^{2+}, had no effect on the PRRT2 inhibition of SNARE N-terminal assembly (Figures S4A and S4B), further signifying that SNAREs are the primary targets of PRRT2.

The topological analysis of PRRT2 shows that PRRT2 is a type II transmembrane protein with a C-terminal single membrane-spanning segment and an N-terminal proline-rich domain that localizes intracellularly (Rossi et al., 2016). So, we reasoned that the N-terminal proline-rich region, which is ideally positioned to interact with the SNAREs on the pre-synaptic membranes, might be the effector domain. This is reinforced by the low-affinity SNARE interaction, a characteristic of polyproline stretches (Kay et al., 2000; Williamson, 1994). To confirm this, we introduced a tobacco etch virus (TEV) protease cleavage site at residue 248 ($PRRT2^{TEV}$) to allow precise *in situ* excision of the N-terminal domain from the membrane anchor

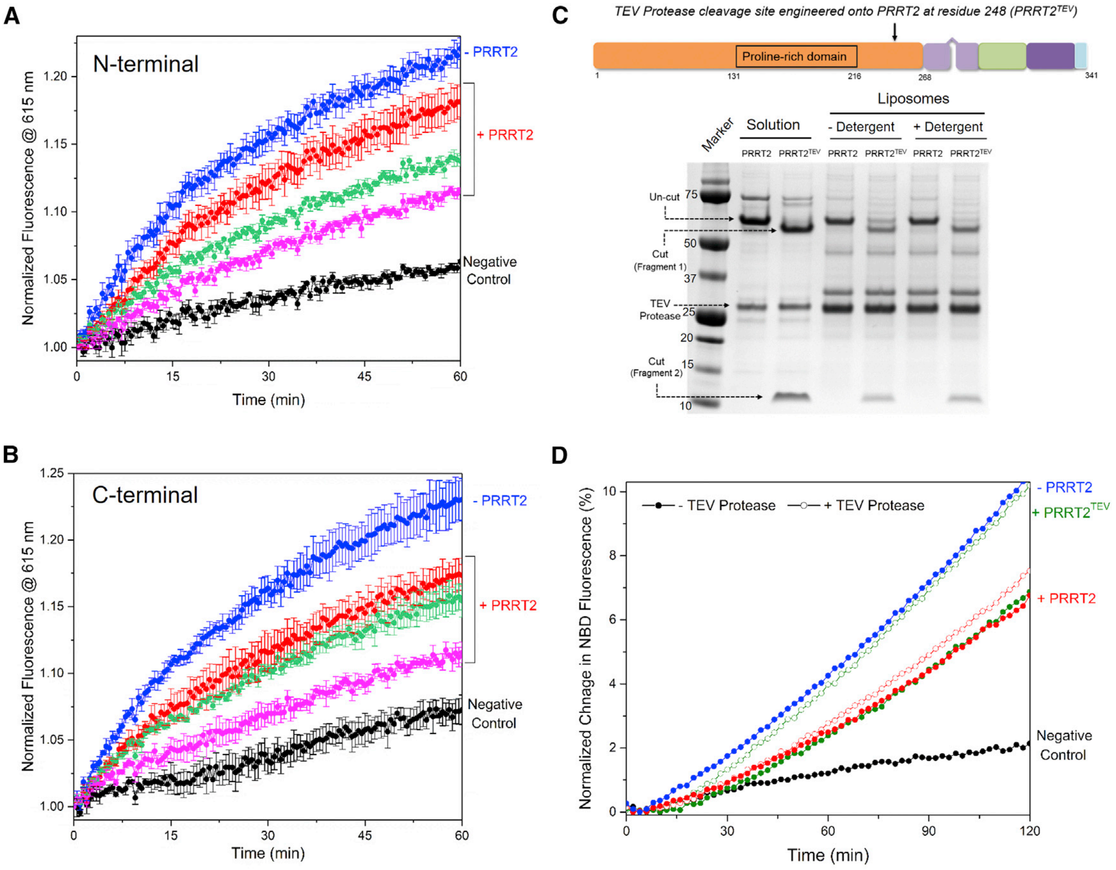

Figure 4. The N-Terminal Proline-Rich Domain of PRRT2 Blocks SNARE Complex Assembly

(A) Assembly of the SNARE complex followed using FRET between Oregon green-labeled t-SNARE and Texas red-labeled VAMP2 introduced in the N terminus (SNAP25 residue 20 and VAMP2 residue 28) shows that PRRT2 blocks the initial engagement of the SNARE complex and does so in a concentration-dependent manner. PRRT2/t-SNARE ratios of 1:1 (red), 2:1 (green), and 3:1 (magenta) are shown. Excess soluble VAMP2 (black) was used as a negative control.

(B) Consistent with the polarized assembly of the SNARE proteins, a similar level of inhibition was observed for different PRRT2/t-SNARE ratios (same color scheme as in A) for the labels introduced in the C terminus (SNAP25 residue 193 and VAMP2 residue 75). Averages and SDs from four independent experiments are shown.

(C and D) PRRT2 contains a large, intracellular N-terminal proline-rich domain (orange), followed by a membrane associated region (light purple) and a transmembrane domain (dark purple), connected by a short, flexible loop (green) (C). *In situ* removal of the PRRT2 N-terminal domain using an engineered TEV protease site (denoted by the arrow) (PRRT2^{TEV}) as confirmed by SDS-PAGE analysis results in complete loss of PRRT2 function in the lipid-mixing fusion assay (D), indicating that the proline-rich region is the effector domain. The fusion curves for PRRT2 and PRRT2^{TEV} with (open) or without (filled) TEV protease treatment are shown. Representative fusion curves and average values and SDs from three or four independent experiments are shown.

(Figure 4C). Reconstituted assay showed the PRRT2^{TEV} blocks fusion effectively prior to the TEV treatment, but this inhibition is completely abrogated following the TEV protease cleavage (Figures 4C and 4D). A control experiment showed that TEV treatment alone does not result in loss of SNARE inhibitory function (Figure 4D). Thus, not only is the N-terminal region required for clamping, but its close positioning on the membrane is essential for its function. Supporting this, the soluble N-terminal proline-rich domain (residues 1–217) and the reconstituted C-terminal portion (residues 209–341) by themselves are unable to produce the clamping effect (Figures S4C and S4D). Thus, PRRT2 inhibitory activity is achieved by interaction of N-terminal proline-rich domain with the individual SNARE proteins, and its proper positioning on the membrane is also crucial for its function.

Paroxysmal Dyskinesia-Associated *PRRT2* Mutations Disrupt the SNARE-Modulatory Function

Given that PRRT2 negatively regulates synaptic fusion, it is easy to imagine how haploinsufficiency and loss-of-function mutations, which can result in unregulated neurotransmitter release, might ultimately lead to paroxysmal neurological disorders. To test this proposition, we focused on a series of *PRRT2* mutations recently identified in a large cohort of 145 families or patients affected with PKD or other paroxysmal neurological disorders (Gardiner et al., 2015). In this study, the consequence of the pathogenic mutations in *PRRT2* were evaluated on both protein and mRNA levels. Also, possible non-sense-mediated decay associated with the (truncating and missense) variants was tested with cDNA sequencing and quantification (Gardiner et al., 2015). A majority of the identified PKD mutations resulted

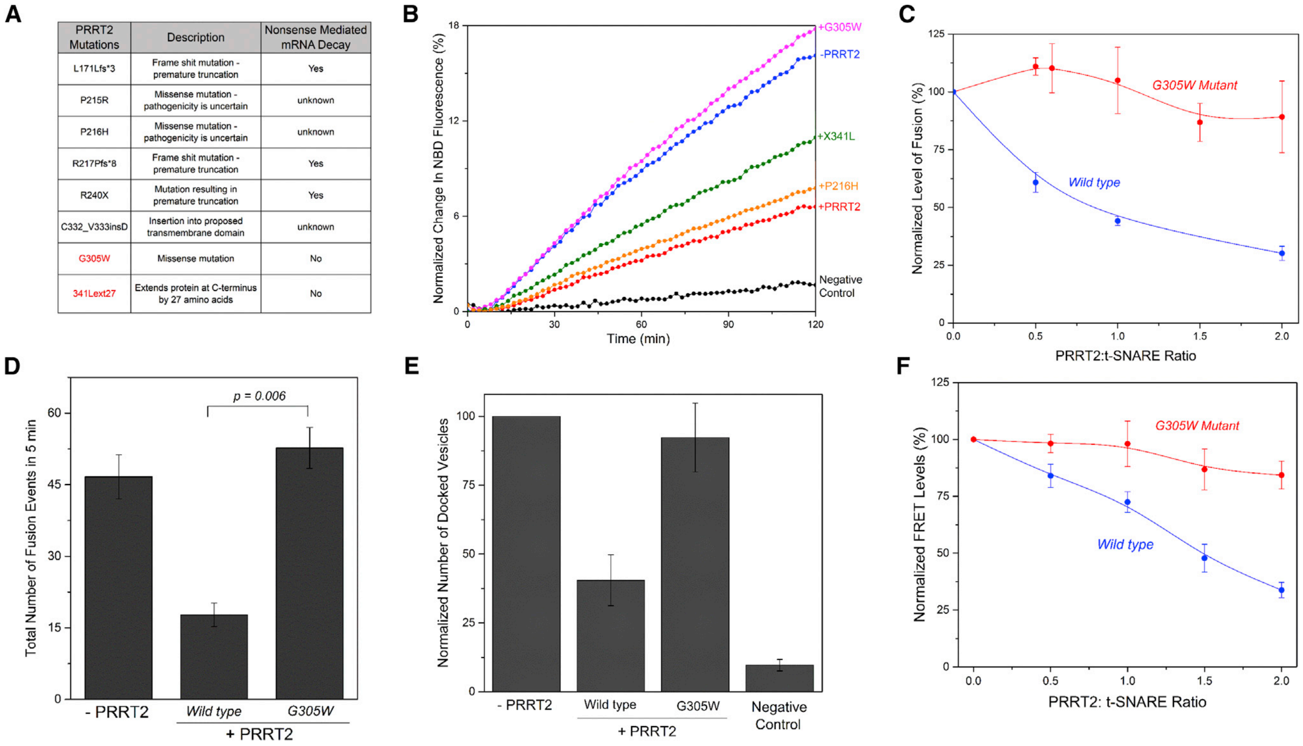

PRRT2 Mutations	Description	Nonsense Mediated mRNA Decay
L171Lfs*3	Frame shit mutation - premature truncation	Yes
P215R	Missense mutation - pathogenicity is uncertain	unknown
P216H	Missense mutation - pathogenicity is uncertain	unknown
R217Pfs*8	Frame shit mutation - premature truncation	Yes
R240X	Mutation resulting in premature truncation	Yes
C332_V333insD	Insertion into proposed transmembrane domain	unknown
G305W	Missense mutation	No
341Lext27	Extends protein at C-terminus by 27 amino acids	No

Figure 5. PKD-Associated Mutations in PRRT2 Disrupt Its SNARE-Modulatory Function

(A) List of PKD-associated mutations in PRRT2 identified by Gardiner et al. (2015). The key mutations (highlighted in red) that did not affect the protein levels and exhibit no non-sense-mediated mRNA decay were tested in this study.

(B) Lipid-mixing assay showing that the PKD mutations G305W (magenta) and X341L (green) disrupt the SNARE-inhibitory function of PRRT2 (red) to different extents. In comparison, the non-pathogenic P216H (yellow) mutation has no effect on PRRT2 function. Negative control with soluble cytoplasmic domain of VAMP2 (CDV) added in excess to titrate out the t-SNAREs is also shown. Representative fusion curves are shown.

(C) Dose analysis shows that the loss of function of G305W mutation (red) in the reconstituted fusion assay is complete and not reversed at a higher dosage of PRRT2. In contrast, the wild-type (blue) PRRT2 shows a typical dose curve. Averages and SDs for three independent trials are shown.

(D) Consistently, the single-vesicle exocytosis assay in PC12 cells shows that the G305W mutation abrogates the SNARE-modulatory function of PRRT2 under physiological conditions. Average values and SDs from a minimum of three independent experiments are shown.

(E) Single-vesicle docking analysis showing that the PKD-associated G305W mutation introduced in the same molar ratio as the wild-type in the v-SNARE liposomes (Figure S5A) does not impede the SNARE-dependent docking/priming of vesicles to the supported bilayers. Average values and SEMs of three to five independent experiments are shown.

(F) FRET-based SNARE assembly assay reveals that the loss of function phenotype results from the inability of G305W mutant (red) to inhibit the SNARE engagement, in contrast to the wild-type (blue) PRRT2. Average values and SDs from three or four independent experiments are shown.

in non-sense-mediated decay; thus, pathogenesis could result from diminished protein levels (Figure 5A). However, two mutations (p.G305W and p.X341L) did not affect the mRNA levels, implying that these mutations primarily alter the protein function (Figure 5A). We therefore used our reconstituted fusion assay to investigate the effect of these point mutations on PRRT2 regulatory function. Both the mutations, reconstituted at the same density as the wild-type (Figure S5A), disrupted the PRRT2 clamping ability but to varying degrees (Figure 5B). The G305W mutation completely abrogated the PRRT2 inhibition, while the X341L (a stop-loss mutation that extends the protein by 27 residues) showed partial loss of function (Figure 5B). As a control, we looked at a non-pathogenic PRRT2 variant (p.P216H), which was found in a British control population (Gardiner et al., 2015). The P216H mutation did not alter PRRT2's ability to block SNARE-mediated fusion, underscoring the specificity of the loss-of-function phenotype observed in the PKD-associated p.G305W and p.X341L mutations (Figure 5B).

It is noteworthy that the severity of the PKD phenotype observed in the mutations corresponded to the magnitude of its effect in the *in vitro* functional analysis. The X341L variant, which presents partial loss of function, was identified in a patient showing typical PKD episodes with short-lasting dyskinesia triggered by sudden movements or exercise. On the other hand, the G305W missense variant, which resulted in complete loss of function, occurred as a *de novo* mutation in a patient with a severe PKD phenotype. This patient has had presented episodes of PKD, usually triggered by excitement or exercise, since the age of 9 years. Interestingly, her presentation and disease course was remarkably more severe compared with other *PRRT2* mutation carriers, with only partial response to

carbamazepine and mild cognitive dysfunction with verbal, memory, and executive function deficits.

Thus, we characterized the G305W mutation in further detail. Dose response using bulk fusion analysis showed that the G305W mutation results in complete loss of function and cannot be rescued by increasing PRRT2 dosage (Figures 5C and S5A). Consistent with this, the mutated PRRT2 also exhibited reduced efficacy in blocking Ca^{2+}-triggered exocytosis in PC12 cells (Figures 5D and S5B), without an effect on the number of PM-adjacent vesicles (Figure S5B). The single-vesicle docking analysis also confirmed the loss of PRRT2 function in moderating SNARE-mediated priming with the G305W mutation (Figure 5E). As expected, FRET analysis showed that the G305W mutation eliminates PRRT2 ability to bind and hinder the SNARE complex assembly, and this deficiency is irreversible (Figures 5F and S5C). Taken together, these data strongly suggest that the pathology of these PKD-associated PRRT2 mutations is directly linked to reduction or loss of its SNARE-regulating function.

DISCUSSION

The data presented here establish that PRRT2 negatively regulates synaptic fusion by directly interfering with the assembly of the SNARE complex. Specifically, it moderates the initial engagement of the cognate SNARE proteins. By doing so, PRRT2 acts to regulate the vesicle priming process, which involves the formation and maintenance of the *trans* SNARE complex, thereby limiting the density of fusion-competent vesicles. This is consistent with the common phenotype observed in all PRRT2 knockdown/KO studies: an accumulation (~2-fold increase) of vesicles at shorter distances to the PM under resting conditions (Tan et al., 2017; Valente et al., 2016) and accelerated replenishment of the readily releasable pool, leading to a higher level of synaptic facilitation (Michetti et al., 2017; Tan et al., 2017).

Purified PRRT2 binds Syt1 with affinity comparable with the SNAREs, but inclusion of Syt1 or Ca^{2+} does not alter PRRT2 inhibitory function in the reconstituted fusion or SNARE assembly analysis. This, taken together with the fact that PRRT2 moderates Ca^{2+}-regulated exocytosis in PC12 cells and has only a modest effect on the efficiency and rate of fusion of pre-docked vesicles, argues against any proposed role for PRRT2 in Ca^{2+} coupling or directly modulating the late stages of vesicle fusion process. PRRT2's role in regulating neurotransmission is still under debate given the conflicting results of the recent *in vivo* studies. Complete loss of PRRT2 either via KO (Michetti et al., 2017) or using a truncation (stop codon) mutation (Tan et al., 2017) exhibits no defect in spontaneous or evoked neurotransmitter release. However, short hairpin RNA (shRNA)-based silencing of PRRT2 knocks down both spontaneous and evoked synchronous release and alters the synchronous/asynchronous release ratio (Valente et al., 2016). Some of this variability could be due to the extensive defects in synaptic structure and organization induced by PRRT2 downregulation (Valente et al., 2016; Valtorta et al., 2016). This could be a consequence of the impaired vesicle trafficking process or hints at yet undefined ancillary PRRT2 role in synaptic development and remodeling. Delineating between these possibilities requires further analysis using more subtle alterations in PRRT2, likely involving *de novo* or engineered mutations.

The data also reveal that PKD-associated mutations disrupt the PRRT2 ability to hinder SNARE assembly and block the resultant membrane fusion. The loss of SNARE-moderating function of PRRT2, which likely dysregulates neurotransmitter release resulting in hyper-excitability, provides a simple and straightforward explanation for the episodic neurological phenotypes presented by the PKD mutation carriers. In fact, this might represent a common mechanism underlying the paroxysmal disturbances associated with loss-of-function PRRT2 mutations. PRRT2 mutations often exhibit pleiotropic effects, with identical mutations showing variable phenotypes and presenting different diseases. This suggests that other factors, including genetic makeup, influence the expression of the disease. Nonetheless, the strong correlation between the presentation and severity of PKD phenotype and the loss of SNARE-modulatory function of PRRT2 highlights the physiological relevancy and the critical nature of this ascribed function.

PRRT2 exerts the fusion block via direct interaction of the N-terminal proline-rich domain with the SNARE proteins. Typically, proline-rich regions mediate multi-valent binding but with weak affinity, as these complexes are not structurally well defined (Kay et al., 2000; Williamson, 1994). In this instance, it appears that the weak interaction of proline-rich region with individual SNARE proteins introduces steric hindrance that impedes productive SNARE assembly. Despite the rather weak affinity measured under soluble conditions, PRRT2 reconstituted alongside either the v- or t-SNARE effectively blocks SNARE assembly and function. It is likely that topological restrictions and orientation imposed by membrane anchoring, combined with the increased local concentration, makes PRRT2 more potent under these conditions. Although the exact concentration of PRRT2 at the synapse is not known, it is widely expressed, and its interaction with the fusion machinery has been verified under *in vivo* conditions (Lee et al., 2012; Tan et al., 2017; Valente et al., 2016). Noteworthy, the weak affinity for assembling SNAREpins might represent a design feature that PRRT2 shares with other synaptic regulators, including Munc13, Munc18, and synaptotagmin, which allows a facile and overlapping control of SNARE complex assembly within the complex and crowded environment of the active zone.

Interestingly, the PKD mutations that were found to cause a severe loss of PRRT2 function (G305W and X341L) without causing non-sense-mediated decay, localize to the C-terminal portion of the protein despite deletion analysis showing that the N-terminal proline-rich region is the primary effector domain. This implies that the C-terminal region also plays a vital role in actuating the SNARE block. It is conceivable that this membrane-proximal, highly conserved amphipathic region interacts with the membranes and/or the SNAREs to position and orient the N terminus to effectively block the initial engagement of the SNARE proteins. This is consistent with our observation that the N-terminal region or C-terminal portion alone is not able to recapitulate a SNARE block. This also highlights the crucial nature of the relative topological configuration of PRRT2 in accomplishing its SNARE-moderating function.

There is limited information on the exact localization of the endogenous PRRT2. Subcellular fractionation analysis shows that PRRT2 is associated with the pre-synaptic area but is diffusely distributed (Tan et al., 2017; Valente et al., 2016; Valtorta et al., 2016). The presently identified PRRT2 role in regulating vesicle docking and the aberrant neurotransmission observed under knockdown or KO conditions (Michetti et al., 2017; Valente et al., 2016) hints at a central role within the active zone. Furthermore, PRRT2 has been shown to bind intersectin1, a scaffold protein involved in the endocytic pathway and concentrated in the periactive zone (Rossi et al., 2016; Valtorta et al., 2016). Further research is needed to identify the precise site(s) of PRRT2 action.

In summary, we have uncovered a crucial role of PRRT2 as a regulator of the synaptic SNARE-mediated release of neurotransmitters at the synapse. Mutations in *PRRT2*, either resulting in changes of the gene reading frame (causing non-sense-mediated decay) or affecting active sites involved in regulating SNARE complex assembly, cause protein loss of function and impaired neurotransmission. These results shed new light in the molecular etiology of paroxysmal neurological disorders associated with abnormal pre-synaptic vesicle exocytosis.

EXPERIMENTAL PROCEDURES

Materials

The human PRRT2 gene and mutants were cloned into pGEX6p-1 (GE Healthcare) using restriction sites BamHI and XhoI. SNARE and synaptotagmin constructs used in the fusion assays have been described previously (Mahal et al., 2002; Melia et al., 2002; Weber et al., 1998). For the PC12 cell-based assay, PRRT2 WT and G305W mutant were cloned into pEF1α-HA (Clontech) using restriction enzymes NcoI and EcoRI. The VAMP2-pHluorin construct has been described previously (Miesenböck et al., 1998). Lipids, 1-palmitoyl-2-oleoyl-snglycero-3-phosphocholine (POPC), 1,2-dioleoyl-sn-glycero-3- (phospho-L-serine) (DOPS), 1,2-dipalmitoyl-snglycero-3-phosphoethanolamine-N-(lissamine rhodamine B sulfonyl) (RHO-DOPE), 1,2-dipalmitoyl-sn-glycero-3-phosphoethanolamine-N-(7-nitro-2-1,3-benzoxadiazol-4-yl) (NBD-DOPE), and ATTO647-DOPE were purchased from Avanti Polar Lipids.

Protein Purification

Synaptotagmin I and all SNARE proteins were expressed and purified as previously described (Melia et al., 2002; Tucker et al., 2004; Weber et al., 1998). The GST-PRRT2 was purified similarly with small modifications. PRRT2 was expressed in *E. coli* strain Rosetta2(DE3) (Novagen), and cells were lysed by cell disruptor (Avestin) in buffer containing 25 mM HEPES (pH 7.4), 400 mM KCl, 4% Triton X-100, 10% glycerol, 0.5 mM Tris(2-carboxyethyl)phosphine hydrochloride (TCEP), and 1 mM phenylmethylsulfonyl fluoride (PMSF). Samples were clarified using a 45Ti rotor (Beckman Coulter) at 140,000 × *g* for 30 min and incubated with glutathione agarose (Pierce) overnight at 4°C. Resin was washed in the same buffer with 1% octylglucoside (OG) and the protein cleaved off the resin using 100 U of PreScission Protease (GE Healthcare) overnight at 4°C. The concentration was determined using a Bradford Assay (BioRad) with BSA as a standard. Protein purity was verified using SDS-PAGE analysis, with Coomassie stain, and identity was confirmed by western blot using a PRRT2 antibody (Atlas). Both showed a single band at ∼60 kDa, corresponding to native protein in the P2 synaptosomal fraction.

Proteoliposome Reconstitution and Bulk Fusion Assay

Proteoliposomes containing SNAREs with or without PRRT2 were prepared using rapid detergent (1% octylglucoside) dilution and dialysis, followed by float-up using a discontinuous Nycodenz gradient as previously described (Ji et al., 2010; Weber et al., 1998). The lipid composition was 85% POPC, 15% DOPS, and the final protein/lipid ratio was 1:200 for VAMP2, 1:400 for t-SNARE, and 1:800 for synaptotamin1. PRRT2 was included into the v- or t-SNARE liposome sample as indicated at the desired ratio during reconstitution. The assembled proteoliposomes were diluted, and vitrified samples were analyzed by cryoelectron microscopy for their appearance and quality. The reconstitution efficiency for both SNAREs and PRRT2 were nearly identical (50%–60%), and thus input ratios are denoted in all dose-dependence analysis. A high input concentration of PRRT2 (more than two copies per SNARE) lowered the SNARE reconstitution efficiency, and thus, in order to attain higher ratios, PRRT2 reconstituted into both v- and t-liposomes was used. Liposome fusion assays were performed as previously described at 37°C using a SpectraMax M5 (Molecular Devices) plate reader, and the fluorescence signal was normalized using maximum fluorescence observed after addition of detergent (2.5% [w/v] n-dodecylmaltoside [DM]).

Single-Vesicle Fusion Analysis

Single-vesicle fusion measurements were performed on a free-standing lipid bilayer as described previously (Kuhlmann et al., 2017) with some recently described hardware and software modifications (S.K.R., A. Gohlke, F. Li, J.C., X. Wu, J.E.R., and F. Pincet, unpublished data). Briefly, free-standing lipid bilayers were formed from t-SNARE-containing giant unilamellar vesicles (GUVs) prepared using the osmotic shock protocol described recently (Motta et al., 2015). Typically, ∼100 nM (total lipid concentration) of VAMP2-containing vesicles (1:400 protein/lipid ratio, ±PRRT2 at 1:2 molar ratio and 2% ATTO647N-DOPE) were introduced into the chamber, and a laser scanning confocal microscope equipped with a 488 nm argon laser and a 647 nm diode laser was used to track the association and fusion of individual vesicles. All experiments were performed at 37°C, and images were acquired at a speed of 68 ms. The images were then analyzed using custom-made software (S.K.R., A. Gohlke, F. Li, J.C., X. Wu, J.E.R., and F. Pincet, unpublished data) to automatically detect and classify the stages of vesicle docking/undocking and fusion. To measure the overall fusion levels in an unbiased manner (Figure 1), all vesicles irrespective of tethering/docking status were considered. To explicitly focus on the late stage of membrane fusion (Figure 3), only vesicles that were firmly docked on the membrane without any detachment in the image sequences before fusion were chosen for the analysis. The PRRT2/t-SNARE ratio is not discernable under these conditions, as the t-SNAREs can freely diffuse on the planar bilayer.

Live Cell Imaging

PC12 cells were incubated at 37°C in 5% CO_2 in RPMI 1640, supplemented with 10% horse serum, 5% FBS, MEM non-essential amino acid, 1 mM sodium pyruvate, and 100 μg/mL penicillin/streptomycin (GIBCO). Cells were transferred to Optimem (GIBCO) with 5 μg of Vamp2-pHluorin plasmid DNA and 10 μg of pEF1α-HA, HA-PRRT2, or HA-PRRT2-G305W and then transfected using a NEPA21 Electro-Kinetic electroporator. The transfection and protein expression were verified by western blot analysis using a PRRT2 antibody. For imaging analysis, cells were plated on collagen-4-coated 50 mm glass-bottomed dishes (Mattek) and imaged 48 hr later. Medium was replaced with Live Cell Imaging Solution (GIBCO), and cells were imaged at 37°C using a custom-made TIRF microscope (Rivera-Molina and Toomre, 2013; Xu et al., 2011) using an Olympus IX-70 inverted microscope fitted with a 60×, 1.45 numerical aperture (N.A.). TIRF microscopic lens (Olympus) and controlled by μManager (https://micro-manager.org). Excitation of pHluorin was done by the 488 nm line of an Innova 70C-Spectrum ion laser (Coherent). Cells were imaged at 6.6 Hz with 150 ms exposure and detected with a Zyla 4.2 megapixel Camera (Andor Technologies). The emission is collected through a BrightLine full-multiband laser filter set, optimized for 405, 488, 561, and 635 nm laser sources (part number LF405/488/561/635-A-000; Semrock). Single-vesicle exocytosis events were automatically detected using MATLAB software (The MathWorks) described previously (Sebastian et al., 2006; Toomre, 2012; Xu et al., 2011). The statistical significance of the fusion results was established using a Wilcoxon-Mann-Whitney test to account for the non-normal distribution of the data using the open-source software Gnumeric (http://www.gnumeric.org).

EM Analysis

Cultured PC12 cells on coverslips were fixed in 2.5% glutaraldehyde in 0.1 M sodium cacodylate buffer (pH 7.4) at room temperature (RT) for 1 hr. The cells

were post-fixed in 0.5% osmium tetraoxide (OsO_4) at RT for 30 min, followed by another 30 min in 1% tannic acid solution. Specimens were stained en bloc with 2% aqueous uranyl acetate for 15 min, dehydrated in a graded series of ethanol to 100%, and embedded in Polybed 812 resin. Blocks were polymerized in a 60°C oven for 24 hr. Thin sections (60 nm) were cut by a Leica ultramicrotome and post-stained with 2% uranyl acetate and lead citrate. Cell sections were examined with a FEI Tecnai transmission electron microscope at 80 kV accelerating voltage; digital images were recorded with an Olympus Morada charge-coupled device (CCD) camera and iTEM imaging software. Images were analyzed using ImageJ to measure the distance between the PM and the dense core vesicles.

Single-Vesicle Docking Analysis

Single-vesicle docking experiments were carried out on supported lipid bilayers as described previously (Karatekin and Rothman, 2012; Xu et al., 2016) with slight modifications. Supported bilayers were prepared by incubating t-SNARE liposomes (~60 μL of 2 mM total lipid) with 5 mM $MgCl_2$ on an Ibidi glass-bottom channel μ-slide for 40 min. The homogeneity and the fluidity of every prepared supported lipid bilayer, after extensive wash with Mg^{2+}-free buffer, was verified using NBD-DOPE fluorescence and by fluorescence recovery after photo-bleaching (FRAP), respectively (Figure S3). For docking analysis, ~100 nM of v-SUVs prepared with VAMP2-4X (VAMP2 with L70D, A74R, A81D, and L84D) and ATT0-647-DPPE ± PRRT2 were allowed to dock on the membranes for 10 min and then extensively washed (20 chamber volume) to remove non-specifically bound SUVs. Approximately 10–12 pictures were collected at different locations of the chip with an inverted Nikon Ti-E total internal reflection (TIRF) microscope equipped with 63X TIRF oil immersion objective (N.A. = 1.45), 647 nm diode laser, and Andor iXon electron-multiplying CCD (EMCCD) camera controlled by NIS-Elements software. Images were projected onto the CCD chip at a magnification of 0.17 μm/pixel with 1.5× intermediate magnification. For an unbiased particle count, we used a custom-written algorithm to count particles from top left to bottom right that ensures every spot is counted only once. The PRRT2/t-SNARE ratio is not discernable under these conditions.

FRET Assay

For site-specific labeling with fluorophores, cysteines were introduced into t-SNAREs (SNAP25 residues 20 and 193) and VAMP2 (residues 28 and 75) using the Quickchange (Stratagene) Mutagenesis kit. Thiol-reactive fluorescent probes Oregon green 488 maleimide and Texas red C5 bromoacetamide (Life Technologies) were used to label SNAP25 and VAMP2, respectively, as described previously (Krishnakumar et al., 2013). We ensured that the labeling efficiency was >75% in all cases, and the labeled SNARE proteins and unlabeled PRRT2 were reconstituted into liposomes, and the SNARE assembly was followed as described previously (Krishnakumar et al., 2013) using 2.5 μM each of the labeled proteins.

TEV Protease Cleavage Site

Site-directed mutagenesis was used to engineer a TEV protease cleavage site in PRRT2 by adding a six-residue recognition sequence (ENLYFQ) at residue 248. $PRRT2^{TEV}$ protein was purified and reconstituted into lipids as described above. The reconstituted liposomes were pre-treated with TEV protease for 2 hr at RT prior to the start of the fusion assays. SDS-PAGE analysis of both free and liposome-reconstituted protein showed that 2 hr at RT results in near complete cleavage.

SUPPLEMENTAL INFORMATION

Supplemental Information includes five figures and can be found with this article online at https://doi.org/10.1016/j.celrep.2017.12.056.

ACKNOWLEDGMENTS

We thank Dr. Kirill Volynski and Dr. Dimitri Kullmann for critical reading of the manuscript. This work was supported by the Wellcome Trust Strategic Award (104033) to J.E.R. and H.H. This work was also supported by a European Research Council (ERC)-funded grant under the European Union's Horizon 2020 research and innovation program (grant agreement 669612). We acknowledge the use of the Center for Cellular and Molecular Imaging (CCMI) EM Core Facility, supported by the Yale School of Medicine.

AUTHOR CONTRIBUTIONS

J.C., O.J., S.K.R., M.N.Z., and J.W. performed the *in vitro* experiments. J.C., O.J., S.K.R., J.E.R., and S.S.K. designed the experiments, analyzed the data, and wrote the manuscript. V.S. and H.H. collected, analyzed, and wrote the clinical data. All authors read and edited the manuscript.

DECLARATION OF INTERESTS

The authors declare no competing interests.

Received: July 4, 2017
Revised: November 12, 2017
Accepted: December 17, 2017
Published: January 16, 2018

REFERENCES

Bello, O.D., Auclair, S.M., Rothman, J.E., and Krishnakumar, S.S. (2016). Using ApoE nanolipoprotein particles to analyze SNARE-induced fusion pores. Langmuir *32*, 3015–3023.

Brose, N., Petrenko, A.G., Südhof, T.C., and Jahn, R. (1992). Synaptotagmin: a calcium sensor on the synaptic vesicle surface. Science *256*, 1021–1025.

Burgoyne, R.D., and Morgan, A. (1998). Analysis of regulated exocytosis in adrenal chromaffin cells: insights into NSF/SNAP/SNARE function. BioEssays *20*, 328–335.

Chen, W.J., Lin, Y., Xiong, Z.Q., Wei, W., Ni, W., Tan, G.H., Guo, S.L., He, J., Chen, Y.F., Zhang, Q.J., et al. (2011). Exome sequencing identifies truncating mutations in PRRT2 that cause paroxysmal kinesigenic dyskinesia. Nat. Genet. *43*, 1252–1255.

Delcourt, M., Riant, F., Mancini, J., Milh, M., Navarro, V., Roze, E., Humbert-claude, V., Korff, C., Des Portes, V., Szepetowski, P., et al. (2015). Severe phenotypic spectrum of biallelic mutations in PRRT2 gene. J. Neurol. Neurosurg. Psychiatry *86*, 782–785.

Diao, J., Grob, P., Cipriano, D.J., Kyoung, M., Zhang, Y., Shah, S., Nguyen, A., Padolina, M., Srivastava, A., Vrljic, M., et al. (2012). Synaptic proteins promote calcium-triggered fast transition from point contact to full fusion. eLife *1*, e00109.

Ebrahimi-Fakhari, D., Saffari, A., Westenberger, A., and Klein, C. (2015). The evolving spectrum of PRRT2-associated paroxysmal diseases. Brain *138*, 3476–3495.

Gardiner, A.R., Jaffer, F., Dale, R.C., Labrum, R., Erro, R., Meyer, E., Xiromerisiou, G., Stamelou, M., Walker, M., Kullmann, D., et al. (2015). The clinical and genetic heterogeneity of paroxysmal dyskinesias. Brain *138*, 3567–3580.

Geppert, M., Goda, Y., Hammer, R.E., Li, C., Rosahl, T.W., Stevens, C.F., and Südhof, T.C. (1994). Synaptotagmin I: a major Ca2+ sensor for transmitter release at a central synapse. Cell *79*, 717–727.

Ji, H., Coleman, J., Yang, R., Melia, T.J., Rothman, J.E., and Tareste, D. (2010). Protein determinants of SNARE-mediated lipid mixing. Biophys. J. *99*, 553–560.

Karatekin, E., and Rothman, J.E. (2012). Fusion of single proteoliposomes with planar, cushioned bilayers in microfluidic flow cells. Nat. Protoc. *7*, 903–920.

Kay, B.K., Williamson, M.P., and Sudol, M. (2000). The importance of being proline: the interaction of proline-rich motifs in signaling proteins with their cognate domains. FASEB J. *14*, 231–241.

Krishnakumar, S.S., Radoff, D.T., Kümmel, D., Giraudo, C.G., Li, F., Khandan, L., Baguley, S.W., Coleman, J., Reinisch, K.M., Pincet, F., and Rothman, J.E. (2011). A conformational switch in complexin is required for synaptotagmin to trigger synaptic fusion. Nat. Struct. Mol. Biol. *18*, 934–940.

Krishnakumar, S.S., Kümmel, D., Jones, S.J., Radoff, D.T., Reinisch, K.M., and Rothman, J.E. (2013). Conformational dynamics of calcium-triggered activation of fusion by synaptotagmin. Biophys. J. *105*, 2507–2516.

Kuhlmann, J.W., Junius, M., Diederichsen, U., and Steinem, C. (2017). SNARE-mediated single-vesicle fusion events with supported and freestanding lipid membranes. Biophys. J. *112*, 2348–2356.

Kyoung, M., Zhang, Y., Diao, J., Chu, S., and Brunger, A.T. (2013). Studying calcium-triggered vesicle fusion in a single vesicle-vesicle content and lipid-mixing system. Nat. Protoc. *8*, 1–16.

Lai, Y., Choi, U.B., Leitz, J., Rhee, H.J., Lee, C., Altas, B., Zhao, M., Pfuetzner, R.A., Wang, A.L., Brose, N., et al. (2017). Molecular mechanisms of synaptic vesicle priming by Munc13 and Munc18. Neuron *95*, 591–607.e10.

Lee, H.Y., Huang, Y., Bruneau, N., Roll, P., Roberson, E.D., Hermann, M., Quinn, E., Maas, J., Edwards, R., Ashizawa, T., et al. (2012). Mutations in the gene PRRT2 cause paroxysmal kinesigenic dyskinesia with infantile convulsions. Cell Rep. *1*, 2–12.

Li, F., Pincet, F., Perez, E., Eng, W.S., Melia, T.J., Rothman, J.E., and Tareste, D. (2007). Energetics and dynamics of SNAREpin folding across lipid bilayers. Nat. Struct. Mol. Biol. *14*, 890–896.

Li, F., Kümmel, D., Coleman, J., Reinisch, K.M., Rothman, J.E., and Pincet, F. (2014). A half-zippered SNARE complex represents a functional intermediate in membrane fusion. J. Am. Chem. Soc. *136*, 3456–3464.

Li, F., Tiwari, N., Rothman, J.E., and Pincet, F. (2016). Kinetic barriers to SNAREpin assembly in the regulation of membrane docking/priming and fusion. Proc. Natl. Acad. Sci. U S A *113*, 10536–10541.

Ma, C., Su, L., Seven, A.B., Xu, Y., and Rizo, J. (2013). Reconstitution of the vital functions of Munc18 and Munc13 in neurotransmitter release. Science *339*, 421–425.

Mahal, L.K., Sequeira, S.M., Gureasko, J.M., and Söllner, T.H. (2002). Calcium-independent stimulation of membrane fusion and SNAREpin formation by synaptotagmin I. J. Cell Biol. *158*, 273–282.

Malsam, J., Parisotto, D., Bharat, T.A., Scheutzow, A., Krause, J.M., Briggs, J.A., and Söllner, T.H. (2012). Complexin arrests a pool of docked vesicles for fast Ca2+-dependent release. EMBO J. *31*, 3270–3281.

Melia, T.J., Weber, T., McNew, J.A., Fisher, L.E., Johnston, R.J., Parlati, F., Mahal, L.K., Sollner, T.H., and Rothman, J.E. (2002). Regulation of membrane fusion by the membrane-proximal coil of the t-SNARE during zippering of SNAREpins. J. Cell Biol. *158*, 929–940.

Michetti, C., Castroflorio, E., Marchionni, I., Forte, N., Sterlini, B., Binda, F., Fruscione, F., Baldelli, P., Valtorta, F., Zara, F., et al. (2017). The PRRT2 knockout mouse recapitulates the neurological diseases associated with PRRT2 mutations. Neurobiol. Dis. *99*, 66–83.

Miesenböck, G., De Angelis, D.A., and Rothman, J.E. (1998). Visualizing secretion and synaptic transmission with pH-sensitive green fluorescent proteins. Nature *394*, 192–195.

Motta, I., Gohlke, A., Adrien, V., Li, F., Gardavot, H., Rothman, J.E., and Pincet, F. (2015). Formation of giant unilamellar proteo-liposomes by osmotic shock. Langmuir *31*, 7091–7099.

Rivera-Molina, F., and Toomre, D. (2013). Live-cell imaging of exocyst links its spatiotemporal dynamics to various stages of vesicle fusion. J. Cell Biol. *201*, 673–680.

Rossi, P., Sterlini, B., Castroflorio, E., Marte, A., Onofri, F., Valtorta, F., Maragliano, L., Corradi, A., and Benfenati, F. (2016). A novel topology of proline-rich transmembrane protein 2 (PRRT2): hints for an intracellular function at the synapse. J. Biol. Chem. *291*, 6111–6123.

Ryan, T.A. (2001). Presynaptic imaging techniques. Curr. Opin. Neurobiol. *11*, 544–549.

Sebastian, R., Diaz, M.E., Ayala, G., Letinic, K., Moncho-Bogani, J., and Toomre, D. (2006). Spatio-temporal analysis of constitutive exocytosis in epithelial cells. IEEE/ACM Trans. Comput. Biol. Bioinform. *3*, 17–32.

Shen, J., Tareste, D.C., Paumet, F., Rothman, J.E., and Melia, T.J. (2007). Selective activation of cognate SNAREpins by Sec1/Munc18 proteins. Cell *128*, 183–195.

Shi, L., Howan, K., Shen, Q.T., Wang, Y.J., Rothman, J.E., and Pincet, F. (2013). Preparation and characterization of SNARE-containing nanodiscs and direct study of cargo release through fusion pores. Nat. Protoc. *8*, 935–948.

Söllner, T., Whiteheart, S.W., Brunner, M., Erdjument-Bromage, H., Geromanos, S., Tempst, P., and Rothman, J.E. (1993). SNAP receptors implicated in vesicle targeting and fusion. Nature *362*, 318–324.

Stelzl, U., Worm, U., Lalowski, M., Haenig, C., Brembeck, F.H., Goehler, H., Stroedicke, M., Zenkner, M., Schoenherr, A., Koeppen, S., et al. (2005). A human protein-protein interaction network: a resource for annotating the proteome. Cell *122*, 957–968.

Südhof, T.C. (2013). Neurotransmitter release: the last millisecond in the life of a synaptic vesicle. Neuron *80*, 675–690.

Südhof, T.C., and Rothman, J.E. (2009). Membrane fusion: grappling with SNARE and SM proteins. Science *323*, 474–477.

Tan, G.H., Liu, Y.Y., Wang, L., Li, K., Zhang, Z.Q., Li, H.F., Yang, Z.F., Li, Y., Li, D., Wu, M.Y., et al. (2017). PRRT2 deficiency induces paroxysmal kinesigenic dyskinesia by regulating synaptic transmission in cerebellum. Cell Res. Published online October 20, 2017. https://doi.org/10.1038/cr.2017.128.

Toomre, D. (2012). Generating live cell data using total internal reflection fluorescence microscopy. Cold Spring Harb. Protoc. *2012*, 439–446.

Tucker, W.C., Weber, T., and Chapman, E.R. (2004). Reconstitution of Ca2+-regulated membrane fusion by synaptotagmin and SNAREs. Science *304*, 435–438.

Valente, P., Castroflorio, E., Rossi, P., Fadda, M., Sterlini, B., Cervigni, R.I., Prestigio, C., Giovedì, S., Onofri, F., Mura, E., et al. (2016). PRRT2 is a key component of the Ca(2+)-dependent neurotransmitter release machinery. Cell Rep. *15*, 117–131.

Valtorta, F., Benfenati, F., Zara, F., and Meldolesi, J. (2016). PRRT2: from paroxysmal disorders to regulation of synaptic function. Trends Neurosci. *39*, 668–679.

Verhage, M., and Sørensen, J.B. (2008). Vesicle docking in regulated exocytosis. Traffic *9*, 1414–1424.

Wang, J.L., Cao, L., Li, X.H., Hu, Z.M., Li, J.D., Zhang, J.G., Liang, Y., San-A, Li, N., Chen, S.Q., et al. (2011). Identification of PRRT2 as the causative gene of paroxysmal kinesigenic dyskinesias. Brain *134*, 3493–3501.

Weber, T., Zemelman, B.V., McNew, J.A., Westermann, B., Gmachl, M., Parlati, F., Söllner, T.H., and Rothman, J.E. (1998). SNAREpins: minimal machinery for membrane fusion. Cell *92*, 759–772.

Westerink, R.H., and Ewing, A.G. (2008). The PC12 cell as model for neurosecretion. Acta Physiol. (Oxf.) *192*, 273–285.

Williamson, M.P. (1994). The structure and function of proline-rich regions in proteins. Biochem. J. *297*, 249–260.

Xu, Y., Rubin, B.R., Orme, C.M., Karpikov, A., Yu, C., Bogan, J.S., and Toomre, D.K. (2011). Dual-mode of insulin action controls GLUT4 vesicle exocytosis. J. Cell Biol. *193*, 643–653.

Xu, W., Nathwani, B., Lin, C., Wang, J., Karatekin, E., Pincet, F., Shih, W., and Rothman, J.E. (2016). A programmable DNA origami platform to organize SNAREs for membrane fusion. J. Am. Chem. Soc. *138*, 4439–4447.

Cell Reports, Volume *22*

Supplemental Information

PRRT2 Regulates Synaptic Fusion by Directly Modulating SNARE Complex Assembly

Jeff Coleman, Ouardane Jouannot, Sathish K. Ramakrishnan, Maria N. Zanetti, Jing Wang, Vincenzo Salpietro, Henry Houlden, James E. Rothman, and Shyam S. Krishnakumar

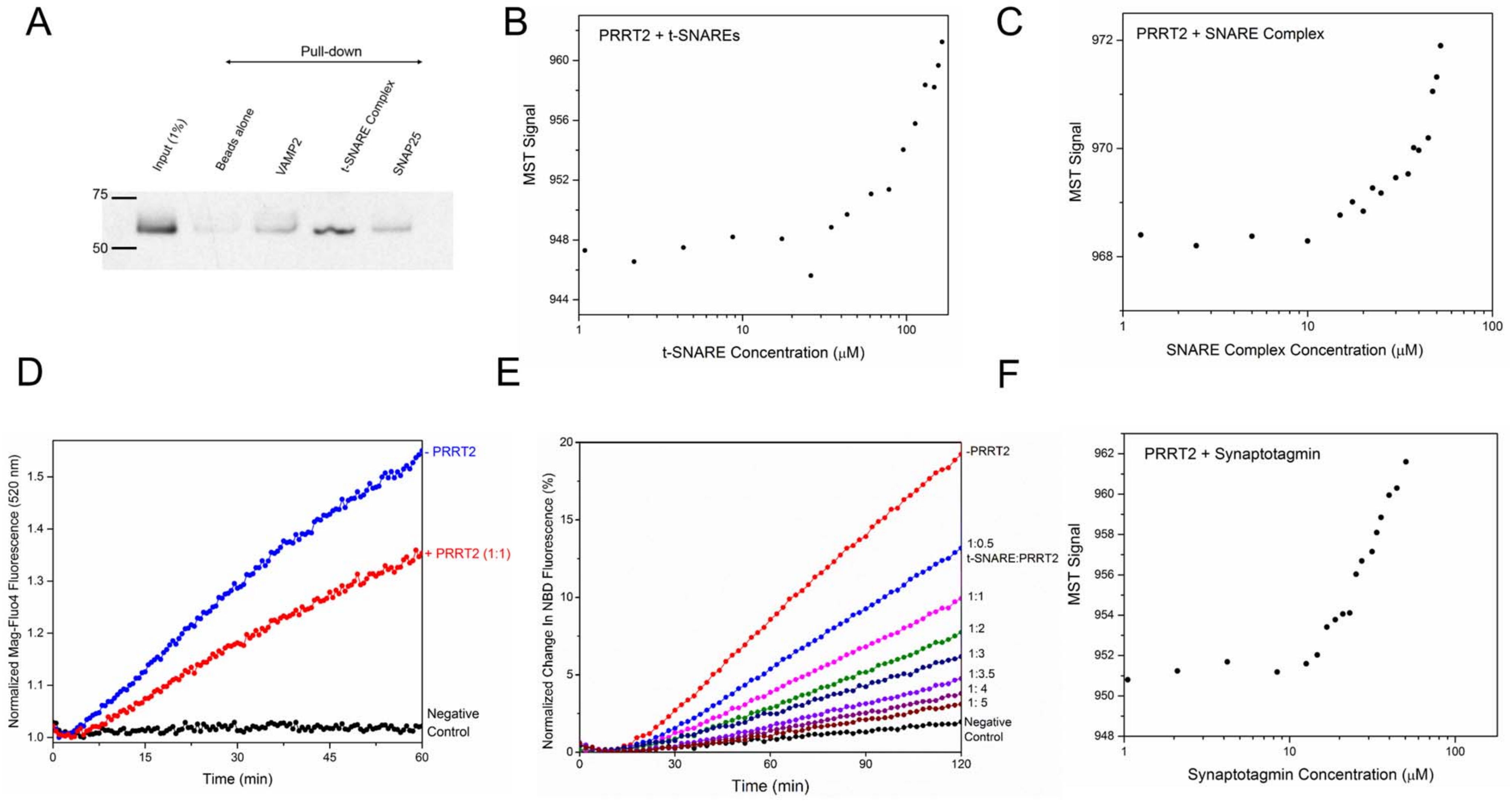

Figure S1. PRRT2 binds individual SNARE proteins to block vesicle fusion

(Related to Figure 1)

PRRT2 has weak interactions with the individual SNARE proteins & Synaptotagmin1. (A) Recombinant PRRT2 was incubated with His^6-tagged individual SNARE proteins (SNAP25 & VAMP2) or assembled t-SNARE (Syntaxin/SNAP25) complex and pulled down using Protein-G resin coated with a monoclonal His antibody. Western blot analysis with PRRT2 antibody revealed the PRRT2 has very weak interaction (1-5% pull-down of the input) with both individual SNARE proteins and assembled t-SNARE complex. PRRT2 interaction with the t-SNAREs (B), SNARE complex (C) and Syt1 (F) was quantified using Microscale Themrophoresis (MST; NanoTemper, Munich, Germany). Themrophoretic properties of PRRT2 labeled at endogenous cysteines with Oregon Green mixed with increasing concentrations of soluble t-SNAREs, pre-assembled SNARE complex and Syt1 was measured using standard treatment capillaries following manufactures instructions. Even at the highest concentration tested (~150 µM for t-SNARE and ~60 µM for SNARE complex and Syt1), the binding was not saturated. But in all cases, we observed binding signal around 25-50 µM underscoring the weak interaction of PRRT2 with the SNAREs and Synaptotagmin. (D) PRRT2 blocks full fusion events. To check this, we adapted a previously described experimental setup (Bello et al., 2016; Shi et al., 2013) using calcium-loaded t-SNARE vesicles fusing with VAMP2-containing nanodiscs, with a calcium-sensitive fluorophore, Mag-Fluo-4 included in the external medium to monitor the release of cargo through a SNARE induced fusion pore. We used larger ~23 nm ApoE-derived nanodiscs containing VAMP2 (vNLPs) and when these vNLP fuses with the calcium-loaded t-SUVs, the Ca^{2+} diffuses through the resulting fusion pore into the exterior buffer, with a consequent increase in Mag-Fluo-4 fluorescence (blue curve). PRRT2 included in the vNLPs at 1:1 molar ratio reduced the Ca^{2+} efflux by ~45% (red curve) confirming that PRRT2 blocks full fusion events. (E) PRRT2 dose analysis using the NBD dequenching liposome fusion assay shows that PRRT2 blocks fusion in a concentration-dependent manner. Increasing amounts of PRRT2 protein were incorporated into t- or v-proteoliposomes, or both in order to reach the indicated ratios of t-SNARE: PRRT2. As a negative control, soluble VAMP2 (CDV) was added in excess to titrate out the t-SNAREs. A representative dose curve is shown and minimum of 3 independent runs were used to estimate the average and deviations shown in Figure 1F.

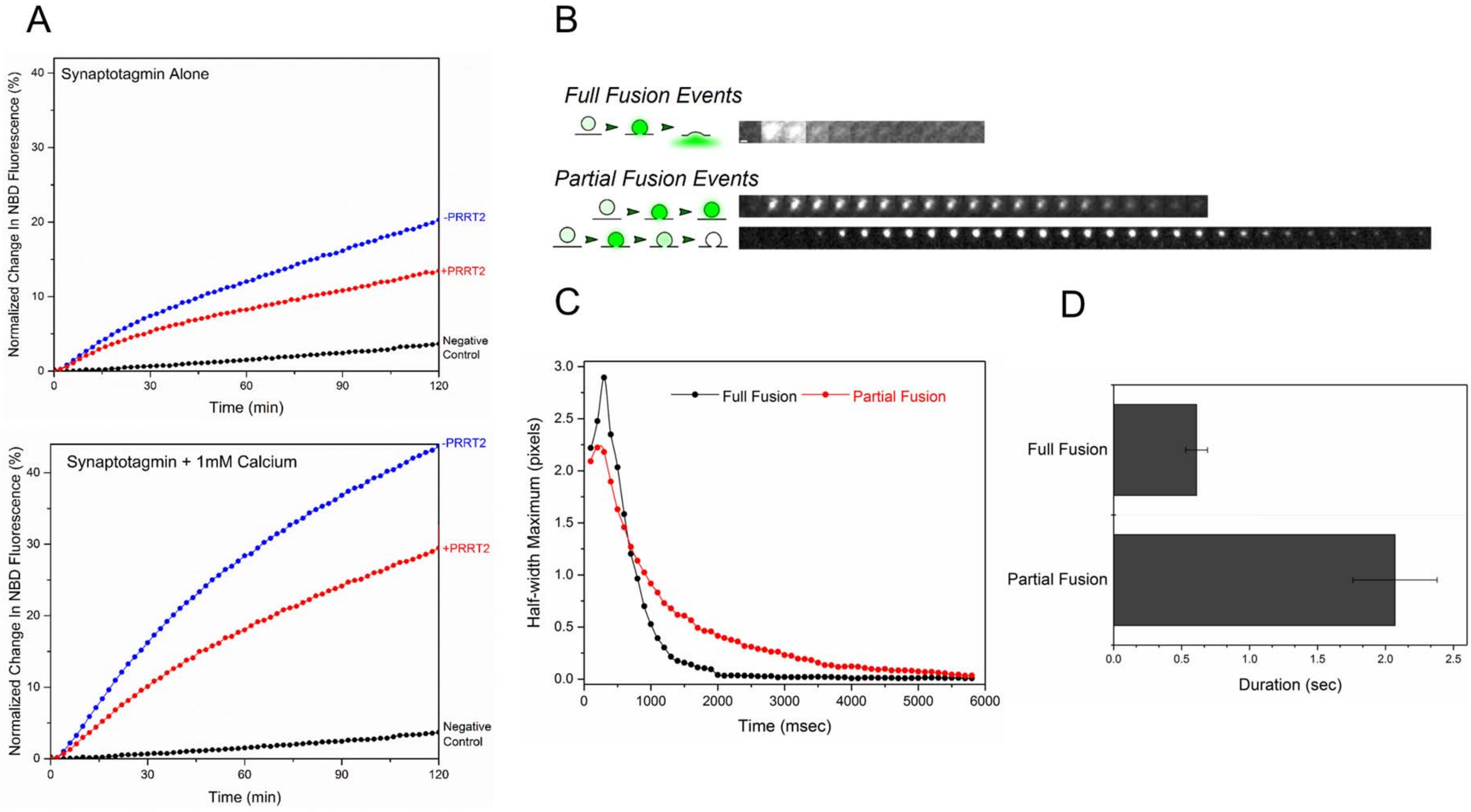

Figure S2. PRRT2 blocks regulated exocytosis under both *in vitro* and *in vivo* conditions.

(Related to Figure 2)

(A) PRRT2 block is realized even in the presence of Synaptotagmin and Ca^{2+}. Lipid mixing assay with PRRT2 and Synaptotagmin reconstituted into t-proteoliposomes (1:1) and v-proteoliposomes (1:4), respectively. The fusion assay was performed with either 0.5 mM EGTA (Synaptotagmin Alone) or 0.5 mM EGTA with 1.5 mM $CaCl_2$ (Synaptotagmin + 1 mM free calcium) in the buffer. In both cases, the inhibitory role of PRRT2 (red curve) was observed and to the same extent indicating that the SNAREs are the primary target of PRRT2 action. Representative fusion curves are shown. (B) Automated analysis of single vesicle exocytosis in PC12 cells using pHluorin under TIRF conditions (A) Time-lapse images of different types of fusion events observed. The fluorescence was recorded at 150 ms per frame under TIRF conditions and cropped to create a time-lapse image. The full fusion events (top) show a diffusive halo and a rapid diffusion of the content, while vesicles undergoing partial fusion (bottom) maintain their shapes and slowly fade due to quenching, slow diffusion through the fusion pore or by leaving the TIRFM area (kiss-and-run events) (C) The fluorescence signal from the vesicle secretion are automatically analyzed by fitting a gaussian with the initial sudden signal increase is set as time zero to align the different events. The average of the half-width versus time shows different dynamics of the different fusion events, with a slower signature for partial fusion events (red), as compared to the faster decay for full-fusion events (black). (D) The comparison of the average duration time (n = 25 cells) for different mode of fusion observed. The duration of an event is defined as the time it takes for the fluorescence to come back to background level signal for a given vesicle. The duration of partial fusion event are substantially (2-3 fold) slower compared to the full-fusion events.

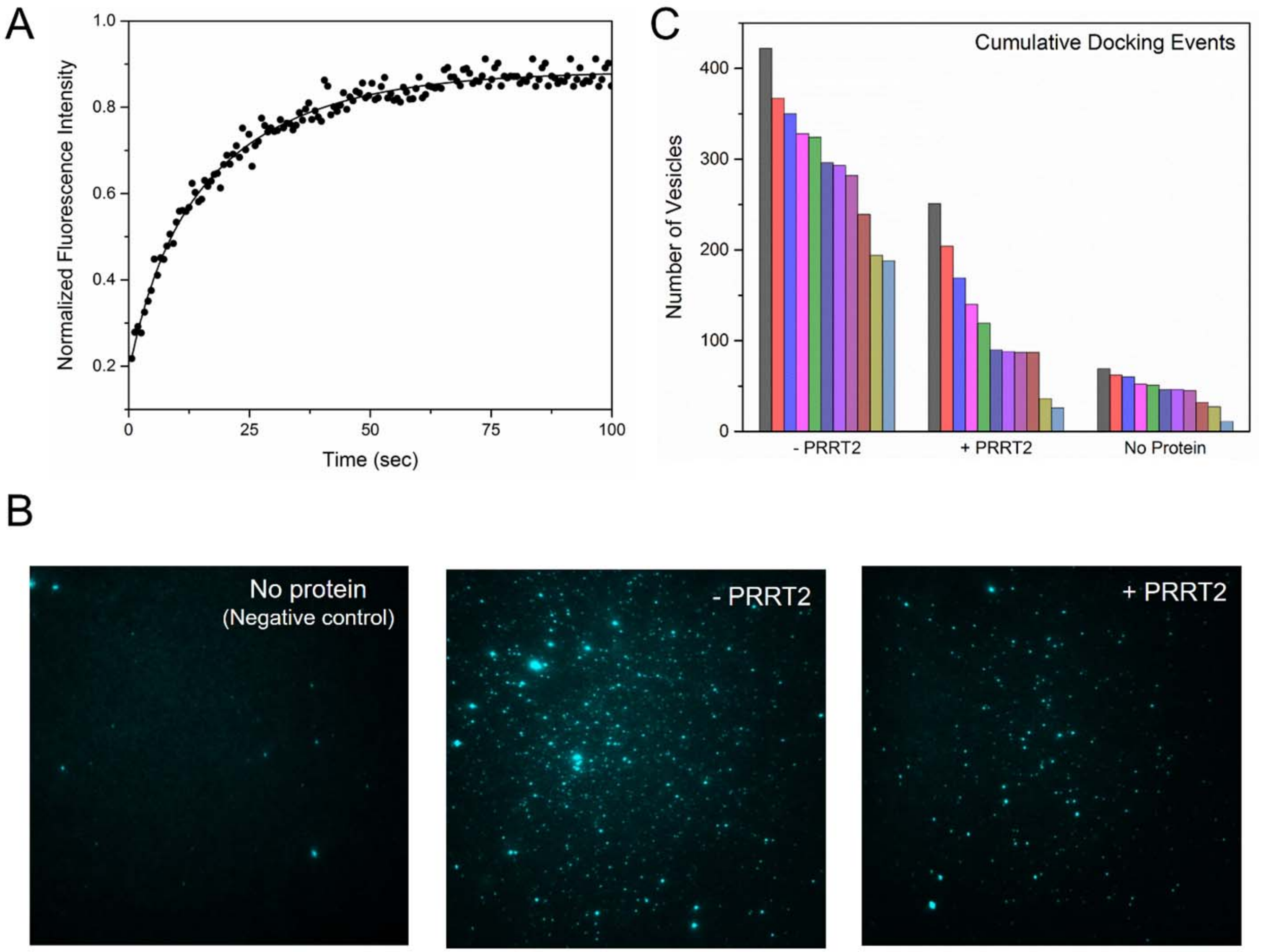

Figure S3. PRRT2 hinders SNARE-mediated docking of individual vesicles to supported bilayer (Related to Figure 3)

The single vesicle docking analysis was carried out under TIRFM conditions, using the ATTO647 dye introduced in the v-liposomes to track the individual vesicles. (A) The fluidity of the t-SNARE containing supported planar bilayer used in the docking analysis was tested by FRAP experiments using the NBD-fluorescence included in the supported bilayer. A representative fluorescence curve (A) shows a rapid recovery following the photo-bleaching consistent with a mobile and fluid bilayer. (B) Representative images of the docked vesicles showing the potent ability of PRRT2 to block SNARE-mediated docking of individual vesicles. (C) Cumulative graph of the number of vesicles docked under various conditions tested. Each color bar denotes an independent experiment carried out with a new supported bilayer. Despite the variability in the total number of vesicles docked between different trials typically introduced by the variability between the supported, bilayers, the trend observed was consistent. For the same concentration of SUVs, there was very little to no docking when VAMP2 was excluded and PRRT2 included in the v-SUVs nearly halved the number of vesicles docking to the supported bilayer.

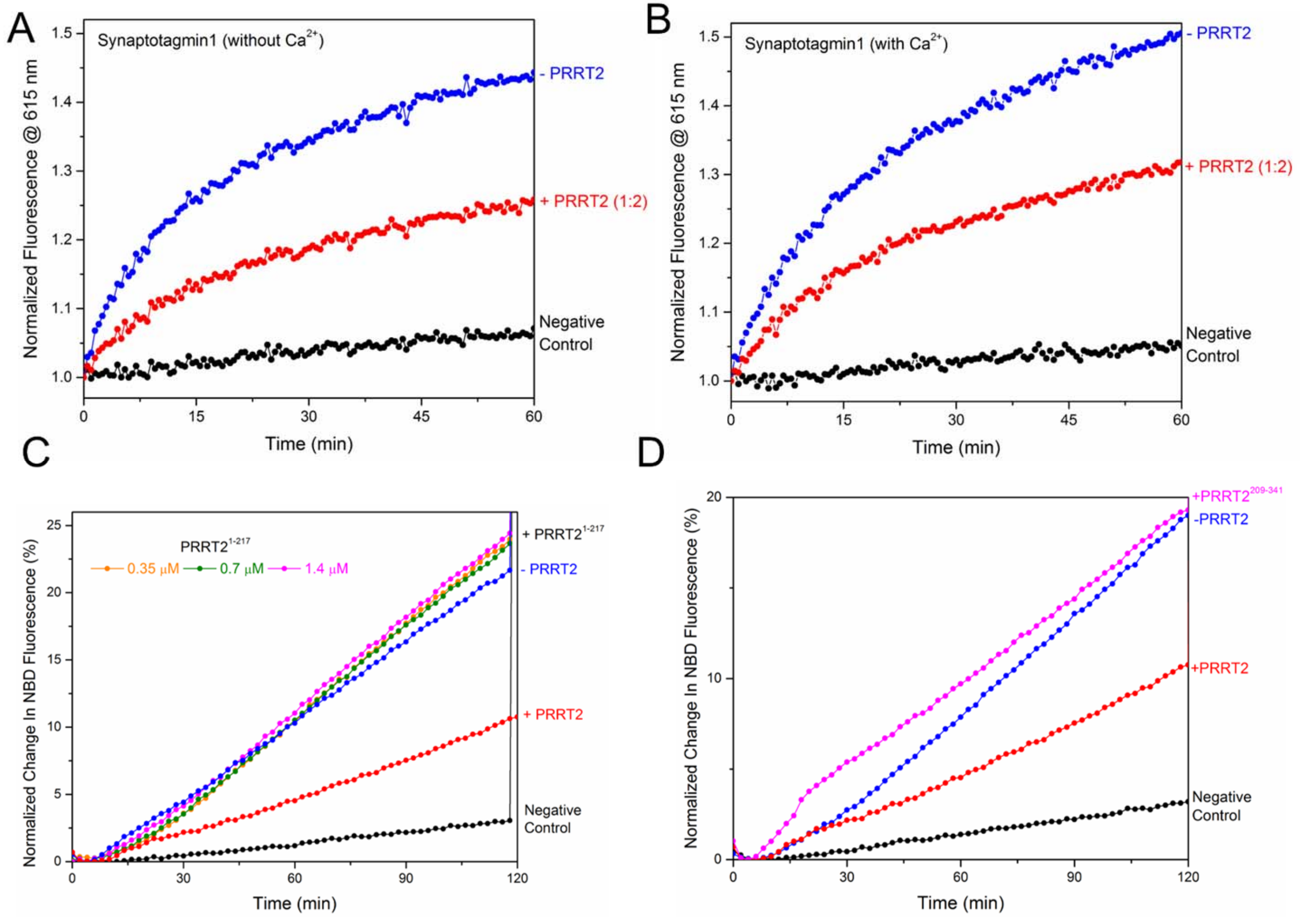

Figure S4. N-terminal Proline-rich domain binds and modulates SNARE Complex Assembly (Related to Figure 4)

(A-B) PRRT2 acts exclusively on the SNARE complex to block fusion. PRRT2 ability to impede the assembly of the N-terminal portion of SNARE complex monitored by FRET between Oregon Green labeled t-SNARE (SNAP25 residue 20) and Texas Red labeled VAMP2 (residue 28) is unaltered by the inclusion of Synaptotagmin1 without Ca^{2+} (A) or with 1 mM free Ca^{2+} (B). Syt1 and Ca^{2+} increases the overall rate and extent of the N-terminal assembly as compared to the SNAREs alone (Figure 4A), but it still inhibited by ~50% when PRRT2 is included in the t-SNARE vesicle at 2:1 PRRT2:t-SNARE ratio. This data confirms that PRRT2-Syt1 interaction is not functionally relevant and SNAREs are the primary target of PRRT2. (C-D) The N- and C-terminal domains of PRRT2 alone are not enough to confer inhibition. (C) The N-terminal portion of PRRT2 (amino acids 1-217) was added to the liposome fusion assay in varying amounts (0.35-1.4 μM) (D) The C-terminal portion (amino acids 209-341) was reconstituted into lipids along with t-SNARE proteins. In both cases, the separate portions of PRRT2 by themselves are unable to inhibit fusion.

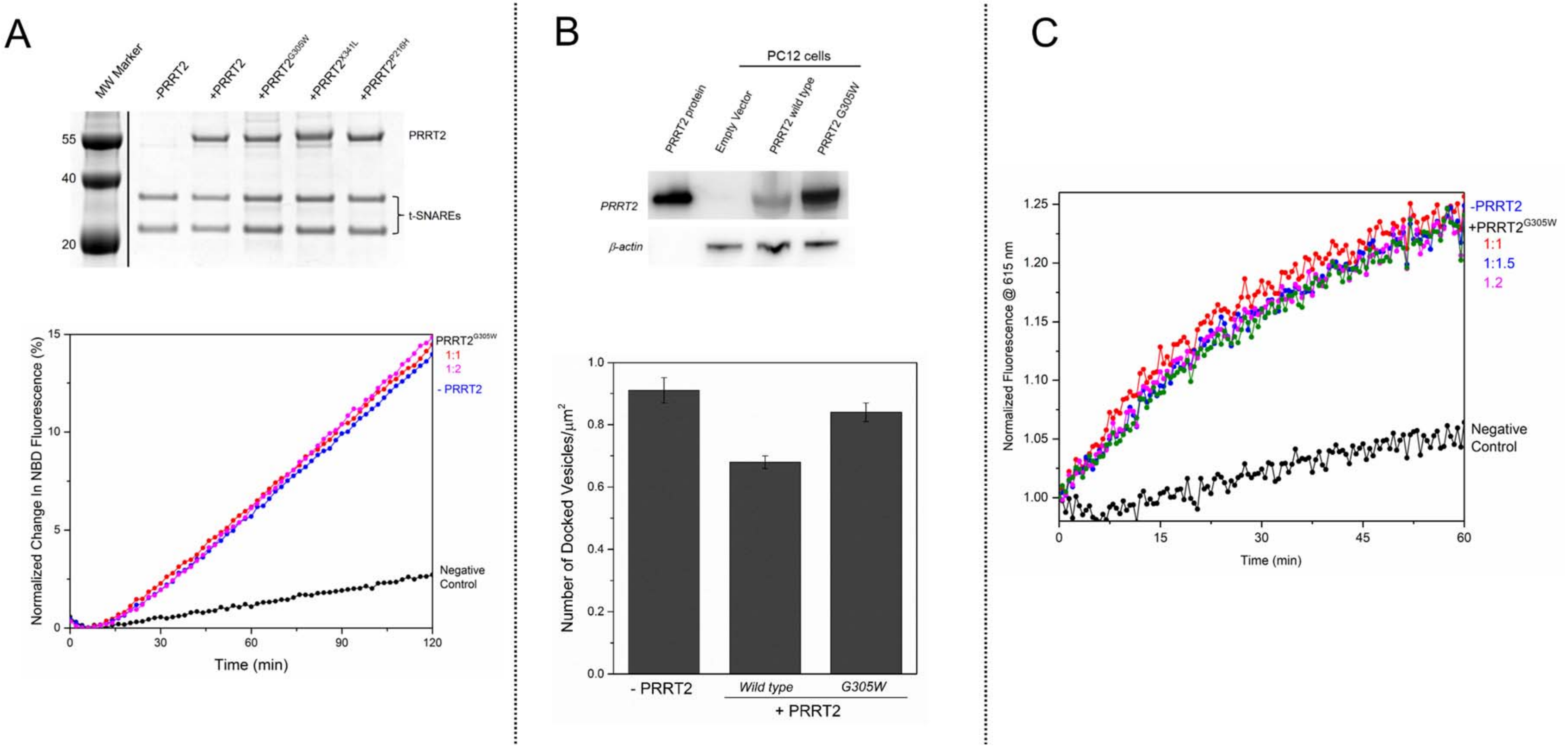

Figure S5. *In vitro* and *In vivo* functional analysis of PKD-associated mutations in PRRT2

(Related to Figure 5)

(A) Coomaisse stained SDS-PAGE analysis of PRRT2 mutants (G305W, X341L & P216H) reconstituted into t-SNARE liposomes. Levels of t-SNAREs were normalized between different samples for liposome fusion assay shown in Figure 5B (top). Dose response PRRT2 mutant $G^{305}W$ in liposome fusion assay at a t-SNARE: PRRT2 ratio of 1:1 and 1:2 shows that the loss of function is not reversed at higher concentrations (bottom). The average and standard deviations from three independent trials are shown in Figure 5C. (B) Characterization of PRRT2 WT and G305W mutant expression and vesicle docking in PC12 cells. Western blot analysis of PC12 cells transfected with PRRT2 WT or G305W showing the lack of endogenous PRRT2 and overexpression of the PRRT2 constructs. A ß-actin was used as a loading control (top). The number of docked vesicles in either wild type or G305W cells as measured by TIRF microscopy following NH_4Cl treatment of the VAMP-pHluorin marker were comparable suggesting that the loss of function phenotype observed in the fusion analysis (Figure 5D) is not due to expression or docking defects (bottom). (C) Monitoring the N-terminal assembly of the SNARE complex using FRET between Oregon Green labeled t-SNARE (SNAP25 residue 20) and Texas Red labeled VAMP2 (residue 28) shows that the G305W mutation disrupts the SNARE modulatory function of PRRT2. Loss of SNARE inhibitory function was observed even at high PRRT2: t-SNARE ratios highlighting the irreversible nature of the G305W mutation. Representative FRET curves are shown and the average and standard deviations on 3-4 independent trials are presented in Figure 5F.

REFERENCES

Bello, O.D., Auclair, S.M., Rothman, J.E., and Krishnakumar, S.S. (2016). Using ApoE Nanolipoprotein Particles To Analyze SNARE-Induced Fusion Pores. Langmuir *32*, 3015-3023.

Shi, L., Howan, K., Shen, Q.T., Wang, Y.J., Rothman, J.E., and Pincet, F. (2013). Preparation and characterization of SNARE-containing nanodiscs and direct study of cargo release through fusion pores. Nat Protoc *8*, 935-948.
